# A Uniquely Stable Trimeric Model of SARS-CoV-2 Spike Transmembrane Domain

**DOI:** 10.3390/ijms23169221

**Published:** 2022-08-17

**Authors:** Elena T. Aliper, Nikolay A. Krylov, Dmitry E. Nolde, Anton A. Polyansky, Roman G. Efremov

**Affiliations:** 1 Shemyakin-Ovchinnikov Institute of Bioorganic Chemistry, Russian Academy of Sciences, 16/10 Miklukho-Maklaya St., 117997 Moscow, Russia; 2 National Research University Higher School of Economics, 101000 Moscow, Russia; 3 Department of Structural and Computational Biology, Max Perutz Labs, University of Vienna, Campus Vienna BioCenter 5, A-1030 Vienna, Austria; 4 Moscow Institute of Physics and Technology (State University), 141701 Dolgoprudny, Russia

**Keywords:** structure prediction, viral fusion protein, molecular dynamics simulation, molecular hydrophobicity potential, transmembrane domain, helical trimer, template-based modeling, Monte Carlo conformational search

## Abstract

Understanding fusion mechanisms employed by SARS-CoV-2 spike protein entails realistic transmembrane domain (TMD) models, while no reliable approaches towards predicting the 3D structure of transmembrane (TM) trimers exist. Here, we propose a comprehensive computational framework to model the spike TMD only based on its primary structure. We performed amino acid sequence pattern matching and compared the molecular hydrophobicity potential (MHP) distribution on the helix surface against TM homotrimers with known 3D structures and selected an appropriate template for homology modeling. We then iteratively built a model of spike TMD, adjusting “dynamic MHP portraits” and residue variability motifs. The stability of this model, with and without palmitoyl modifications downstream of the TMD, and several alternative configurations (including a recent NMR structure), was tested in all-atom molecular dynamics simulations in a POPC bilayer mimicking the viral envelope. Our model demonstrated unique stability under the conditions applied and conforms to known basic principles of TM helix packing. The original computational framework looks promising and could potentially be employed in the construction of 3D models of TM trimers for a wide range of membrane proteins.

## 1. Introduction

The spike (S) protein, crucial to the infectivity of SARS-CoV-2 and other coronaviruses, is a class I viral fusion protein, alongside a number of proteins that have long been under scrutiny, including HIV’s gp41 and haemagglutinin from influenza virus. Like other fusogens of this kind, the S-protein is trimeric and consists of a voluminous ectodomain exposed on the virion surface, an α-helical transmembrane domain (TMD) and a small endodomain [1]. Apart from its well-documented role in receptor recognition, the spike, or, more specifically, its S2 subunit, is a factor effectuating membrane fusion, bringing together the viral and target membranes, facilitating the release of the viral genome into the target cell. To accomplish this function, fusogenic proteins employ their regions tailored to interact with the membranes of both the viral and host cell—TMD(s) and one or several membrane-active fragments such as the fusion peptides [2,3]. Therefore, to understand all stages of viral fusion, one would require exhaustive knowledge of the TMD’s structural subtleties, as they directly contribute to spike protein refolding and the merging of the membranes.

Little experimental information on the SARS-CoV-2 spike TMD (S-TMD) structure has been obtained so far. Recently, a trimerization study has been conducted [4] for a peptide corresponding to SARS-CoV-2’s spike residues 1209–1237, in which Met and Cys residues were substituted for Leu and Ser, respectively, amounting to a total of four point mutations compared to the wild type protein. In dimyristoylphosphatidylcholine/1,2-Dihexanoyl-sn-Glycero-3-Phosphocholine (DMPC/DH_6_PC) bicelles, this peptide assumed a trimeric structure with a Leu/Ile-zipper-like interface. The portion of the peptide whereof the structure was resolved and which spanned the bicelle membrane was mapped as residues 1218 to 1234, corresponding to a rather short TMD fragment 16 residues long (PDB ID 7LC8). Immediately downstream of its ectodomain, the spike contains a small region rich in aromatic residues (1212-WPWYIW-1217), followed by a hydrophobic region (1218-LGFIAGLIAIVMVMTIML-1234). It has previously been shown in an NMR-based study [5] that a peptide corresponding to the C-terminal portion of the stem region followed by the aromatic cluster of the spike in SARS-CoV (residues 1193 to 1202), whereof the spike protein S2 subunit is highly homologous to that of SARS-CoV-2, has the propensity to assume a helix-loop-helix conformation in dodecylphosphocholine micelles, the loop roughly coinciding with the area around I1210 and K1211 (SARS-CoV-2 numbering). Four clusters of at least two cysteines can be found downstream of the TMD, the first one located immediately after it and consisting of Cys 1235 and Cys1236. These residues have been shown to be palmitoylated, and this modification is believed to be crucial to fusion, as the replacement of these residues with alanine resulted in a considerable loss of infectivity in both SARS-CoV-2 [6] and SARS-CoV [7].

Despite experimental structural data on the organization of the S-TMD being scarce, several attempts have been made to model it *in silico*. A number of full-length models of SARS-CoV-2’s spike protein have been built, within which the TMD was reconstructed using template-based modeling. However, it was on no occasion the primary focus of modeling efforts, and the finer, more intricate aspects of its structure might have been overlooked. In one of these models, created by Casalino et al. [8], a water-soluble coiled-coil from a bacterial protein was used as the template, although transmembrane (TM) α-helices are known to possess a primary structure only peculiar to them to guarantee optimum packing tailored to the lipid environment [9,10]. This model only includes palmitoyl modifications at Cys1240 and Cys1241, known as the second cysteine cluster. Shortly afterward, another model became available [11], within which the TMD had been built via homology modeling using NMR data obtained for the TMD from HIV’s gp41, another class I fusion protein and hence the spike’s close functional counterpart. The same structure was used for the template-based modeling of S-TMD in a recent *in silico* study of allosteric effects in the spike [12]. However, it was not examined in detail to what extent the physico-chemical properties of the TM helix were similar in the two fusion proteins. Nor has it been revealed whether the proposed model is in agreement with the known principles of helical TMD organization, namely its structural aspect as well as the distribution of hydrophobic/hydrophilic and conservative/variable residues between the lumen of the trimer and lipid-exposed surface. The models available for download do not include palmitoyl modifications; however, the authors used CHARMM-GUI [13] to add palmitoyl tails to Cys1236 and Cys1241 to perform MD simulations of full-length spike protein binding to its receptor and antibodies. Other models have been derived relying on various automated tools such as Rosetta and I-TASSER as opposed to template-based modeling [14,15,16]; however, it is not possible to evaluate their overall quality at this time.

Like in the case of other integral membrane proteins, the main problem in deciphering the spatial structure of TMDs of viral fusogens is that they have to be stabilized in a native-like membrane environment, while much more voluminous domains of the spike need to stay in the water. Moreover, in the immediate vicinity of the TMD, there are several very conformationally flexible membrane-active regions, which can hamper efforts to obtain a high-resolution structure using X-ray crystallography or cryo-EM techniques. From the computational point of view, the problem lies in the absence of reliable instruments for the prediction of TM homotrimer 3D structure. In the present study, we explore the possibility of solving this task *in silico* for the TMD of SARS-CoV-2’s spike protein solely based on the amino acid sequence. Our main objective was to construct such a model taking into account all the known principles of TM helix packing identified so far. To accomplish this, we designed an original computational framework that is described below.

The design of the present study was as follows:

1. At the first stage, the boundaries of S-TMD were identified via sequence analysis and further ascertained via Monte Carlo (MC) simulations of a TM monomer in an implicit membrane. Eventually, the fragment chosen to model S-TMD consisted of residues 1212 to 1234.
2. At the next stage, an optimal structural template was selected among homotrimers of TM helices with known structures. The template had to conform to the following two criteria: (1) the presence of patterns of residues with physicochemical properties in the TM sequence sufficiently similar to those in the fragment of interest in the spike; (2) a good correspondence of the surface geometric and MHP properties between the two TM segments (in the template and spike). In the absence of sequence homology with available potential templates, the main assumption was that TM helices in S-TMD would pack in a similar way as in a template whereof the monomers possess similar sequence/surface motifs. To this end, we scrutinized the TM sequence of the spike and compared it to all TM homotrimers in the PDB database in search of common patterns of charged, polar, hydrophobic, small, and proline residues. For the candidates thus identified, we compared spatial distributions of hydrophobic/hydrophilic properties on their solvent-accessible surface and selected the optimal one(s).
3. To enhance the reliability of S-TMD modeling, we used an independent approach. Based on MHP complementarity for TM helices, we predicted dimerization interfaces for the TM segment to identify surfaces likely to be on the helix-helix interfaces and compared them against those in the template(s) to pick the optimum one, with helix–helix contact areas as similar to those predicted for S-TMD as possible.
4. One of the candidate templates, the TMD of TNFR-1, was chosen to build a model of S-TMD via homology modeling. To test the stability and viability of this model, MD simulations of this trimer were performed in a model POPC bilayer. POPC was chosen as it mimics, to a sufficient extent, the thickness and composition of the membrane of the endoplasmic reticulum Golgi intermediate compartment (ERGIC), where SARS-CoV-2 virions acquire their envelopes. Additionally, MD simulations of the original template were performed to evaluate its behavior in a POPC bilayer. Based upon MD data, a new, better-packed model trimer (*S_OPT*) was built via iterative adjustment of the ‘dynamic MHP portraits’ of the interacting helices.
5. The stability of model trimer *S_OPT* and several alternative ones was then evaluated via MD simulations (see Table 1 and Table 2).
6. Finally, palmitoyl tails were added at C1235 and C1236 in the *S_OPT* model to explore how such modifications could affect the stability of S-TMD and the way it was accommodated by the membrane during MD simulations.

## 2. Results

### 2.1. S-TMD Boundaries Identified via Sequence Analysis and Monte Carlo Simulations

Different TMD boundaries were predicted with recourse to TMSEG (TMD residues: 1214–1238) and TMPRED (1216–1235) or proposed based on data from UniProt entry P0DTC2 (1214–1234), as well as in work by Xia et al. [2] (1213–1237) and Cai et al. [1] (1212–1234). Subsequent adjustment of S-TMD’s boundaries was made using MC simulations of an S-TMD monomer (residues 1208 to 1239) in an implicit membrane. It should be noted that this segment encompasses all the regions mentioned above.

**Figure 1:**
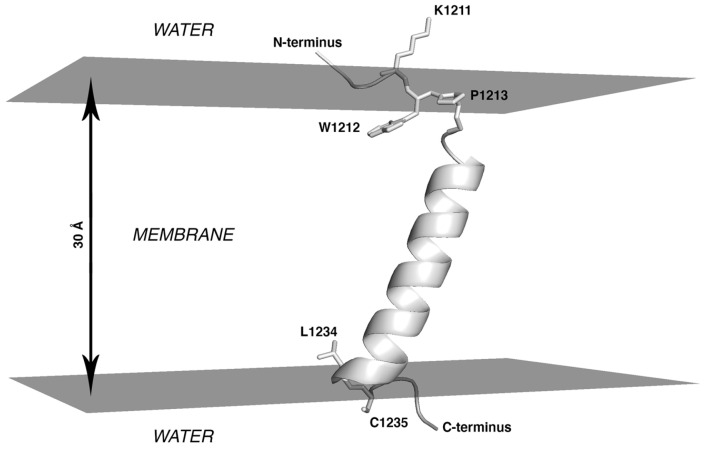
Lowest-energy state for monomeric S-TMD derived via Monte Carlo simulations. Spike fragment 1208–1239 in an implicit membrane is shown. The system is oriented in such a way that the top and bottom surfaces of the hydrophobic slab (here represented in grey) are perpendicular to the Z axis and correspond to XY planes with Z-coordinates of +15Å and −15Å. The protein is shown in cartoon representation with selected residues in stick representation.

Analysis of the conformers accumulated in the course of the MC search invites the following conclusions: (1) Throughout the entire simulation, the peptide preserves its initial α-helical structure well in the portion formed by residues from I1216 to L1234; (2) Being initially placed in the water phase, it partially penetrates into the hydrophobic layer (|Z| < 15 Å) as early as upon preliminary minimization, before the actual MC steps were performed. (3) Subsequent conformational search in the dihedral angles space was accompanied by a decrease of the total energy (*Etot.*) of the system, during which the peptide quickly assumed the TM orientation. (4) The ensemble of the accumulated low-energy MC-states (within 5 kcal/mol from the minimal *Etot.*) only includes the TM segment in the α-helical conformation (residues I1216 to L1234), which spans the hydrophobic layer mimicking the “membrane” (Figure 1). In these states, the angle between the helical axis and the membrane normal (Z-axis) is 25° ± 2°. The peptide is kinked at residue Y1215, while its N-terminal part (residues 1208 to 1215) is disordered. In these states, residues I1210 and W1212 to Y1215 are buried in the membrane (Figure 1).

This result indicates that in the most energetically favorable MC-states, the entire TMS peptide is embedded into the hydrophobic medium and does not expose the N-terminal fragment from W1212 to Y1215 at the lipid/water interface. A different orientation has been proposed in other studies [4], in which the region between Y1209 and W1217 was considered to be a juxtamembrane domain, while the TM part only included residues L1218 to L1234. MC data, therefore, additionally justify the selection of the entire fragment from W1212 to L1234 to build a model of the S-TMD trimer. This choice is also supported by the fact that K1211 immediately upstream thereof is a charged residue highly unlikely to be part of the TMD, and residues 1235 and 1236 are palmitoylated cysteines, likely to be membrane-proximal but not belonging to the TMD *per se*.

### 2.2. TMD of Tumour Necrosis Factor Receptor-1 as an Optimal Template for S-TMD Modelling

**Figure 2:**
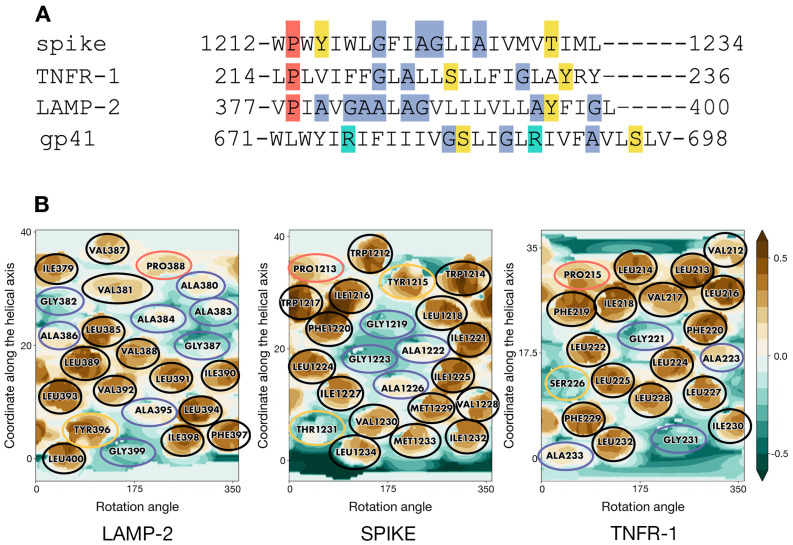
Residue patterns in S-TMD and candidate templates. (**A**) The similarity between patterns in the sequences of spike glycoprotein and TMDs with known 3D structure-LAMP-2 (2MOM), TNFR-1 (7K7A), and gp41 (5JYN). Hydrophobic residues are unhighlighted, while proline, small, charged, and polar residues are highlighted in coral, blue, turquoise, and yellow, respectively. (**B**) Molecular hydrophobicity potential (MHP) distribution maps for an ideal helix corresponding to S-protein residues 1212–1234 and TMD monomers of two candidate templates, LAMP-2 and TNFR-1. Cylindrical projection of the surface MHP distribution is used. Axis values correspond to the rotation angle around the helical axis and the coordinate along the latter (in Å), respectively. An MHP scale (in logP octanol-1/water units) is presented on the right. The maps are colored in accordance with the MHP values (Efremov et al., 1992), from teal (hydrophilic areas) to brown (hydrophobic ones). Projections of proline, small, polar, and hydrophobic residues are encircled in coral, blue, yellow, and black, respectively.

Analysis of sequence patterns formed by polar, hydrophobic, small, and proline residues in a set of homotrimeric TMDs with known 3D structures (Table S1) revealed two structures of interest. Selected as candidate templates for the modeling of S-TMD were lysosome-associated membrane protein 2A (LAMP-2A, PDB ID 2MOM [17]) and tumour necrosis factor receptor-1 (TNFR-1, PDB ID 7K7A [18]). Corresponding sequence alignment is presented in Figure 2A. In spike, TNFR-1 and LAMP-2 one can notice a hydrophobic N-terminal residue followed by a proline and several sporadically located small residues. The polar serine in TNFR-1 was known to not be located on the helix–helix interface, which would therefore be constituted by small and hydrophobic side chains. Close attention was also paid to the TMD of HIV’s gp41 protein [19], a class I viral fusion protein and thus spike’s functional counterpart (PDB ID 5JYN). However, its sequence features two charged arginine residues (Figure 2A), resulting in a pattern different from that in S-TMD, which led us to focus on other candidate templates.

2D MHP maps of monomeric TM helices for the two candidate templates (taken from the corresponding 3D models) and an ideal helix with a sequence corresponding to residues 1212-1234 in S-TMD are shown in Figure 2B. It can be seen that the spike shares a common MHP pattern with TNFR-1: an islet of small residues (G1219, A1222, G1223, A1226 in spike, and G221 in TNFR-1) surrounded by bulkier hydrophobic residues on all sides (Y1215, I1216, L1218, F1220, I1221, L1224, I1225, I1227, V1228, M1229 in spike and V217, I218, F219, F220, L222, L224, L225, L228 in TNFR-1). LAMP-2 has a similar small residue islet (A395 and G399); however, it lacks hydrophobic “lining” C-terminally, as it is immediately followed by the highly polar cytoplasmic domain, while in TNFR-1, this MHP motif is located “higher” up the helix, towards the middle of the TMD. In the light of these observations, we decided to use TNFR-1 as the template in the modeling of S-TMD.

### 2.3. Preliminary Model of S-TMD Homotrimer Based on Predicted Helix–Helix Interfaces

**Figure 3:**
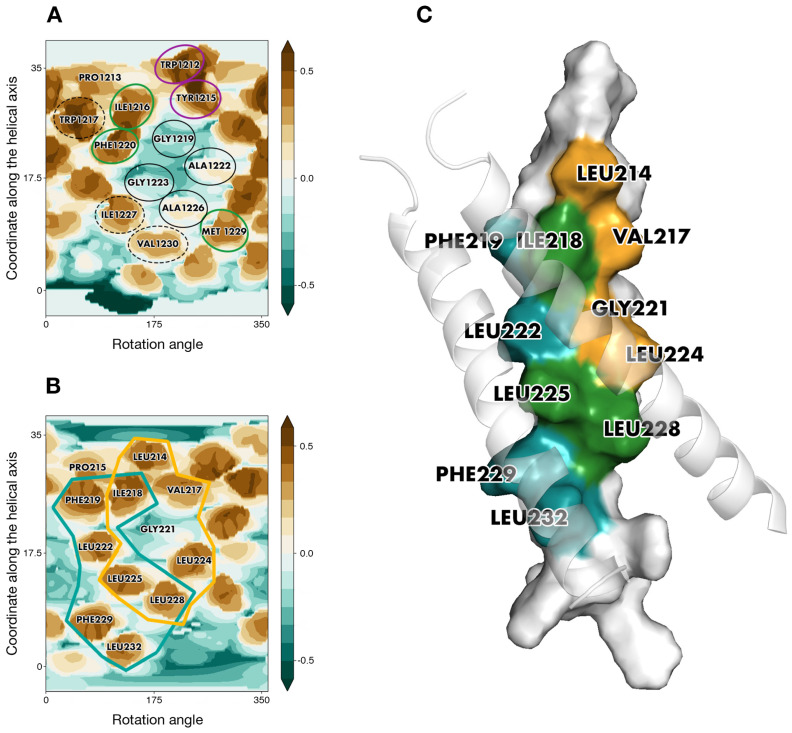
The similarity between S-TMD and a candidate template. (**A**) Interface B is one of the dimerization solutions predicted for S-TMD. (**B**) Overlapping helix–helix interfaces for one of the chains in TNFR-1. TNFR-1’s interface 1 and interface 2 are enclosed in golden and teal lines, respectively. Identical positions are encircled in purple, conservative and semi-conservative residues are encircled in green, non-conservative residues present on the helix–helix interface are encircled in black, and residues in S-TMD that could be part of the helix–helix interface if the latter were similar to that of TNFR-1 are enclosed in dashed lines. For other details, see the legend in Figure 2B. (**C**) A 3D model of the TMD of TNFR-1. Residues that are part of interface 1, part of interface 2, and part of both interfaces are colored golden, teal, and green, respectively. One of the helices is rendered in surface representation, while the other two helices are shown in cartoon representation and are semi-transparent.

Based on MHP complementarity between the surfaces of TM segments, three helix–helix dimerization interfaces were identified for S-TMD and dubbed interfaces A, B, and AB (Figure S1). Amino acid positions in S-TMD were considered identical and (semi-) conservative if they were such among diverse members of genus *Betacoronavirus*. Apart from this, helix–helix interfaces for the TMD of the template TNFR-1 were evaluated using its spatial structure. It turned out to be organized as follows: each helix has two partially overlapping interfaces, interface 1 and interface 2, interacting with either of the two remaining helices. In every helix, interface 1 interacts with interface 2 and vice versa (Figure 3C).

Noteworthily, a pattern formed by W1212, Y1215, I1216, G1219, A1222, G1223, and A1226 in interface B of S-TMD was remarkably redolent of interface 1 identified for TNFR-1 (Figure 3A,B). Furthermore, if the two sequences were aligned for these two interfaces to match, proline residues in TNFR-1 and the ideal helix corresponding to spike’s residues 1212-1234 ended up in highly similar positions in relation to the helix–helix interface. Of equal interest was the fact that the ideal helix with the sequence of S-TMD had additional residues, W1217, F1220, I1227, and V1230, which, together with interface B added up to a pattern we had observed for the candidate template: two partially overlapping interfaces fit to accommodate two helices in a manner similar to that of TNFR-1, which was accordingly chosen for template-based modeling of the spike protein’s TMD.

### 2.4. S_OPT: A Tightly Packed TMD Model Obtained via Iterative Refinement

Some differences were observed between TNFR-1 and our model of the spike protein’s TMD, *S*_*TNFR1*. Most notably, a hydrophobic patch on TNFR-1’s helix–helix interface constituted by one glycine (221) and three leucines (222, 224, 225) corresponded to two alanines (1222, 1226) and two glycines (1219, 1223) in our model. In line with this, the difference in solvent-accessible surface area, or ASA, (dASA) between *S*_*TNFR1* and three helices before assembly equalled ∼1500 Å^2^. In our template, TNFR-1, dASA was nearly twice as high, ∼2950 Å^2^, suggesting that a hollow space was present inside our model, a number of lumen-facing side chains located too far apart to interact and stabilize the trimer and not contributing to helix–helix interface formation (Figure S2D).

In the course of MD simulations of *S*_*TNFR1*, the number of protein–lipid (P/L) contacts grew dramatically, indicating that the helix–helix interface was at least partially exposed to POPC molecules and disrupted by them (Figure S2A). This is barely surprising, considering the imperfections of the trimer derived via direct template-based modeling. In some trajectories, we observed the formation of a symmetrical dimer by two of the three helices of the model with helix–helix interfaces similar to the template’s predicted interface 2 and including I1227 and V1230, confirming that these residues are indeed fit to be located on helix–helix interfaces (data not shown). More interestingly yet, among the resulting MD-states, we found a dimer formed by two of the three helices within the model trimer, whereof residues predicted to constitute interface 1 belonging to one helix interacted with residues predicted to be part of interface 2 belonging to the other helix (Figure S2E). This dimer formed as early as 9 ns and, in the microsecond range, proved to be stable and tightly packed, as is evidenced by RMSD (Figure S2B) and ASA (Figure S2C) dynamics over the course of the MD. dASA for this dimer is estimated at ∼880 Å^2^ (as calculated at 200 ns). At ∼830 ns, however, the dimer underwent a distinct change (Figure S2B) from asymmetrical to roughly symmetrical. After the M1229 in chain C moved away from the interface, so did its W1212, whilst its F1220 ended up facing chain A after slight rotation around the helical axis. Eventually, roughly the same residues in both chains contributed to the helix–helix interface, albeit still undisrupted by lipids. This shift is evident from both the RMSD and ASA dynamics (Figure S2B,C). At the same moment in time (∼830 ns), the average number of pairs of atoms belonging to the side chains on the “initial” asymmetrical interface between helices A and C engaged in protein–protein (P/P) interactions also decreased from 23 to 21.

We thus obtained a structure that could potentially be used to build a *bona fide* trimer, organized in accordance with the same fundamental principles as the TMD of TNFR-1 but without noticeable empty spaces inside the helix bundle. To verify whether this was indeed the case, we manually “cloned” two trimers based on the aforementioned asymmetrical dimer, aligning the “original” dimer and its copy in such a way as to obtain the missing third chain in the predicted position and later deleting the “extra” chain in the copy. The resulting two trimers were exceedingly similar to each other, and one, named *S*_*OPT*, was chosen for further examination.

**Figure 4:**
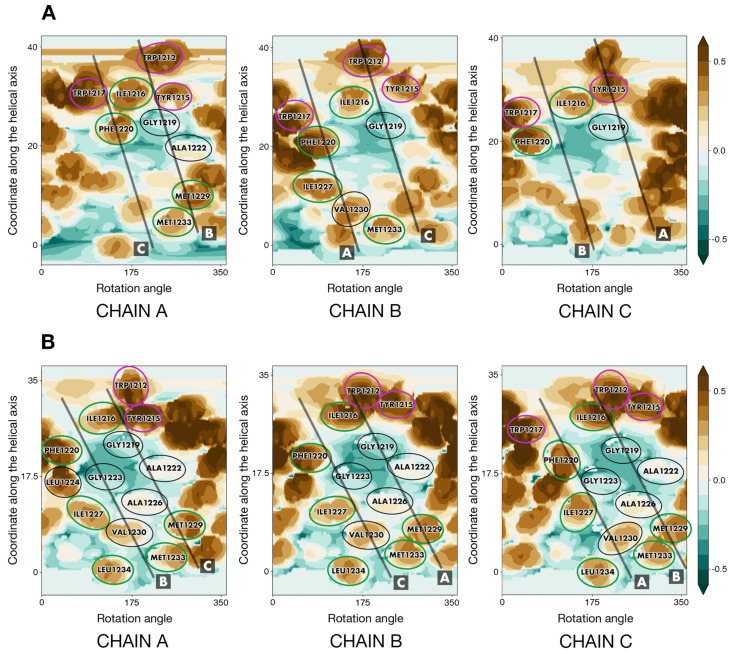
Helix–helix interfaces in *S*_*TNFR1* and *S*_*OPT*. (**A**) Helix–helix interfaces in *S*_*TNFR1* before simulation. (**B**) Helix–helix interfaces in *S*_*OPT* upon building and energy minimization. Identical positions are encircled in purple, conservative and semi-conservative residues are encircled in green, and non-conservative residues present on the helix–helix interface are encircled in black. For each chain, projections of the other two helices are shown as semi-transparent black lines and designated by letters A to C. For other details, see the legend in Figure 2B.

**Figure 5:**
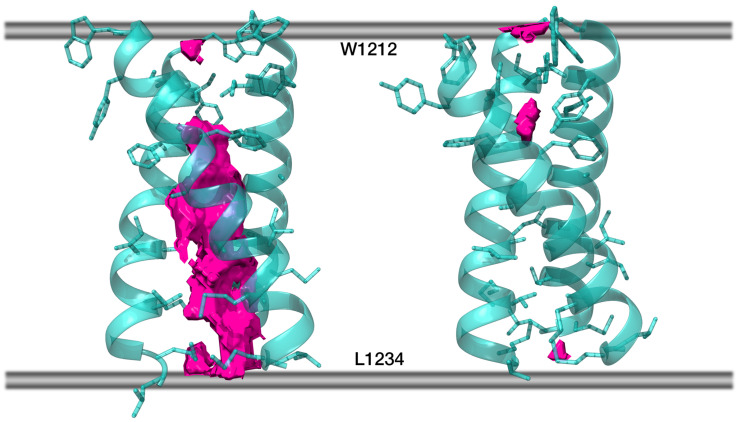
Free volume in the lumen of *S*_*TNFR1* (**left**) and in *S*_*OPT* (**right**). Protein chains are partially transparent and are shown in cartoon representation; residues within each chain facing either of the remaining two chains are shown in stick representation. Free volume is rendered as solid pink blocks (averaging over 200 frames not applied), while the boundaries of the hydrophobic stratum of the membrane are schematically shown as grey lines.

Helix–helix interface maps in *S*_*OPT* were highly similar to those of TNFR-1, as though a post-packing event had occurred during simulation in a bilayer to perfect the structure and squeeze the helices closer together where the hollow space used to be. This space, unstabilized by intermolecular interactions inside *S*_*TNFR1* had been partially located around the area (Figure 4A) constituted by small residues (G1219, A1222, G1223, A1226) in lieu of a bulkier core of three leucines (222, 224, 225) and glycine 221 in TNFR-1. In addition to this, V1230 had also taken the place of leucine residues, failing to fill all of the volumes fit to accommodate bulkier leucine chains. In *S*_*OPT* all of these residues were brought close together so that they ended up interacting (Figure 4B). Furthermore, TNFR-1 had glycine residues at position 231, which in our model corresponded to M1229, more voluminous hydrophobic entities. Together with M1233 and L1234, corresponding to positions in TNFR-1 that are not even part of its transmembrane domain, R235 and Y1236, M1229 formed another tightly packed patch in *S*_*OPT*. This patch fits in well with the “two interfaces per helix, one to interact with either of the remaining two helices” principle, whereby the trimer gains additional turns of the helix contributing to the stabilization of the interface. Much in the same vein, dASA for *S*_*OPT* equalled ∼2580 Å^2^, as opposed to ∼1500 Å^2^ in *S*_*TNFR1*, while the decrease of free volume (FV) observed in the lumen of the trimer in *S*_*OPT* compared to the starting structure is estimated to be approximately 25-fold (Figure 5). The empty space was thus eliminated, as the helices were positioned closer together and engaged in more favorable intermolecular P/P contacts, which stabilized the trimer. Accordingly, in the course of subsequent MD simulations (see Section 2.5 for details) *S*_*OPT* demonstrated much lower RMSD values as compared to *S*_*TNFR1*, amounting to 1.3 ± 0.2 Å for the former and to 2.9 ± 0.4 Å for the latter.

In *S*_*OPT* the distances between the axes of monomers range from 7.8 Å to 10 Å and the angles between helical axes equal 41 ± 2°. Consistently found on the helix–helix interfaces were W1212, I1216, G1219, F1220, A1222, G1223, A1226, I1127, M1229, V1230, M1233, and L1234. Many of these positions (W1212, I1216, F1220, I1227, M1229, M1233, and L1234) can be considered at the very least semi-conservative within genus *Betacoronavirus*, which indicates their functional relevance to a protein encoded by a viral genome with a mutation rate characteristic of ssRNA viruses. Seeing that it was arranged as predicted and contained a remarkable number of (semi-)conserved residues on helix–helix interfaces, a feature often observed in α-helical TMDs [9,20], *S*_*OPT* was selected to test its stability further.

### 2.5. Model S_OPT Is Highly Stable in a Lipid Bilayer: Results of MD Simulations

**Figure 6:**
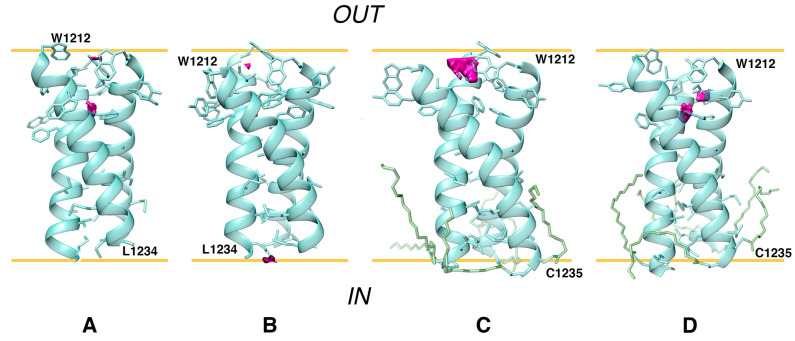
Visualization of free volume in unpalmitoylated and palmitoylated *S*_*OPT*. Free volume in the lumen of the model trimers is rendered as pink blocks (averaging over 200 frames not applied) and shown at 1 μs of MD trajectories *S*_*OPT run1* (**A**), *S*_*OPT run2* (**B**), *S*_*OPT-PLM run1* (**C**), and *S*_*OPT-PLM run2* (**D**). 3D models are shown in cartoon/stick representation; palmitoyl chains are shown in stick representation where present. Approximations of POPC carbonyl/ester oxygen planes are shown as yellow lines. The inside of the virion and the external environment are designated as “IN” and “OUT,” respectively.

For the entire duration of the MD trajectories, the model *S*_*OPT* remained stable: helix–helix interfaces were undisrupted by lipids and retained a high degree of similarity to the model’s initial state (Figure S3A–C). In one of the trajectories, one of the helices moved slightly away from the original interface in its N-terminal portion (W1212/Y1215). Downstream therefrom, however, this chain contributed to the trimer interface as much as the other two. The model trimer, which had initially been oriented along the normal to the membrane plane with residues 1212 to 1234 spanning the bilayer, demonstrated a tendency to remain in the described position, buried in the membrane, and is slightly tilted relative to the normal to the bilayer plane. Indeed, the center of mass of *S*_*OPT*’s residues 1212 to 1234 had a Z-coordinate corresponding to the midpoint between leaflets (Figure S4A,B), while α_ax_, the angle between the trimer principal axis and the normal to the bilayer, equaled 14° ± 7° and 12° ± 5° in trajectories *S*_*OPT run1* and *S*_*OPT run2*, respectively. The number of intermolecular P/P and P/L contacts did not vary significantly over the course of the MD simulations, indicating that the extent of their exposure to the lipid environment did not alter (Table S2). Likewise, evaluation of FV in the lumen of the model TMD over the course of the MD trajectories indicates that it remains tightly packed and whenever unoccupied volume can be detected, the trimer tends to revert back to a tightly packed state (Figure 6 and Figure S4C), while the RMSD oscillations are consistent therewith (Figure S4D).

### 2.6. Dynamic Properties of the Template TNRF-1 TMD and Other S-TMD Models

#### 2.6.1. *TNFR1*_*TMD*

Over the course of a 500 ns MD simulation, the angle α_ax_ amounted to 15 ± 6° (as opposed to 12° at 0 ns). Two stages can be clearly identified in the trajectory (Figure S5). At first, the RMSD of the backbone atoms of the TMD varies very little until, at ∼238 ns, it begins to grow. Similarly, estimated FV in the TMD lumen seems to disappear almost entirely, resulting in tight packing; however, at around 250 ns, it also starts rapidly increasing. At approximately the same moment, side chains constituting the helix–helix interface in *TNFR1*_*TMD* start making an increasingly higher number of contacts with POPC molecules and gradually fewer contacts with each other, indicating that the helix–helix interface was disrupted by lipids. We, therefore, conclude that *TNFR1*_*TMD* was not stable in a model POPC bilayer, possibly partially due to an insufficiently long hydrophobic TM part, as, experimentally, the NMR structure was observed in DMPC/DH_6_PC bicelles and could thus be poorly adapted to a POPC bilayer. Indeed, immediately downstream of the proposed TMD is a flurry of polar and charged residues that require a hydrophilic environment.

#### 2.6.2. Other S-TMD Structures

Three models based on the recently published NMR TM trimer (PDB ID 7LC8) were tested (Table 1): the trimer structure verbatim (trajectory *S*_*NMR*), the same structure with short disordered fragments added upstream and downstream to stabilize the helices and minimize the “disordering” of our fragment of interest (trajectory *S*_*NMR+*) and 7LC8 with the sequence of the wild type spike protein (trajectory *S*_*NMR-WT*). The angle α_ax_ amounted to 27° ± 11°, 12° ± 6° and 21° ± 8° for *S*_*NMR*, *S*_*NMR+* and *S*_*NMR-WT*, respectively. For all the three trimers, we analyzed the number of contacts between the residues constituting the Leu/Ile zipper proposed by the authors of the experimental study (I1221, I1225, L1229, and L1233) and lipids, as well as the contacts these residues make with their counterparts in neighboring helices. It appears that the number of contacts between Leu/Ile zipper residues within the trimer decreases over time, whereas the number of contacts these residues makes with lipids grows. Thus, larger portions of the side chains became exposed to lipids, indicating that the helix–helix interfaces were disarrayed by POPC molecules, and the Leu/Ile zipper, which had been observed experimentally in DMPC/DH_6_PC bicelles was destabilized, no longer holding the trimer together. Residues that had been initially tucked away into the trimer’s lumen become increasingly more exposed to the lipid environment (Figure S6A,B). In line with this is the estimated FV (Figure S6D,E) in the lumen of each of the three trimers examined. In each trajectory, it dramatically fluctuates over time, but the trimer fails to revert back to a relatively tightly packed state it was in at the outset of the simulation with no lipids on the helix–helix interface. RMSD values (Figure S6C) also indicate that initial configuration lifetimes were short for this group of models. The structure experimentally observed in bicelles and its derivatives were thus not stable in a model POPC bilayer.

Both mutants containing substitutions present in the NMR model, *S*_*OPT-LC* and *S*_*OPT-LS*, remained tightly packed, while the angle α_ax_ equalled 13° ± 7° and 9° ± 4° for *S*_*OPT-LC* and *S*_*OPT-LS*, respectively. The behavior of these trimers did not indicate significant destabilization compared to their precursor with the wild-type amino acid sequence, as evidenced by the RMSD and FV dynamics (Figure S6F,G), as well as by the numbers of intermolecular P/P and P/L contacts (Table S2). We thus observed no significant impact of the substitutions present in the experimentally observed model trimer on the organization of helix–helix interfaces nor on the stability of the model trimer *S*_*OPT*. Therefore, instability in a lipid bilayer of the NMR model of S-TMD with four mutations in the sequence would appear to stem from other factors than these modifications. The reason is most likely to lie in the poor adaptation of the NMR-derived template (e.g., mutual packing of helices in a trimer) to the POPC bilayer. However, as the mutations were introduced into a pre-existing model trimer, one cannot rule out the possibility that they could affect TMD trimerization during assembly in the membrane.

The S-TMD model built by Casalino et al. [8] has completely different helix–helix interfaces (Figure S3D), redolent of a canonical water-soluble coiled-coil, with the most voluminous hydrophobic residues tucked inwards facing the trimer lumen. This approach does not take into account the known principles of TM helix packing. We did not explore this model in further detail. Meanwhile, both gp41-based models [11] have helix–helix interfaces very similar to those in *S*_*OPT* (Figure S3E,F) and dASA of 2450 Å^2^/2406 Å^2^. RMSD for the backbone atoms in residues 1212 to 1234 in these two trimers was 2.1 Å and 2.5 Å as compared to *S*_*OPT*. We tested them for stability in a POPC bilayer using the same fragment as in the case of *S*_*OPT* (1208–1238), and their performance was comparable to that of our model insofar as parameters like FV in trimer lumen were similar, not exceeding 10 Å^3^, and consistent throughout the MD simulations. Our model did somewhat better as far as RMSD goes, however: 1.3 ± 0.2 Å and 1.2 ± 0.3 Å for *S*_*OPT* as opposed to 2.9 ± 0.7 Å and 2.4 ± 0.2 Å for the two gp41-based models. Finally, model *S*_*OPT* is better packed based on dASA values (see above). These data suggest our model’s higher propensity for stability.

### 2.7. Palmitoylation of *S*_*OPT* Preserves Its Unique Stability in a Model Membrane

Over the course of MD simulations, both palmitoylated trimers retained their stability (Figure S4D), and their helix–helix interfaces were mainly similar to those in *S*_*OPT*. The angle α_ax_ for *S*_*OPT-PLM* amounted to 19° ± 7° in run1 and 16° ± 7° in run2. Whenever FV could be observed in a trimer’s lumen, the protein reverted to an extremely tightly packed state (Figure S4C), and the number of P/P and P/L intermolecular contacts also remained consistent over the course of the MD simulations (Table S2). The overall stability of the helix–helix interface appears to be comparable in palmitoylated and unpalmitoylated trimers, as is evidenced by RMSF calculations for each residue within the TMD (Figure S4E). The input of each residue into protein/protein contacts holding the trimer together is also uniform across all replicas calculated for *S*_*OPT* and *S*_*OPT-PLM*. The only deviation of relative magnitude observed in palmitoylated models is a greater input on the part of W1217 (Figure S4F).

Apart from preserving the trimer stability, the introduction of palmitoyl modifications does not cause significant perturbation of the lipid environment as compared to the non-palmitoylated trimer. Thus, the analysis of the order parameter of lipid tails (S_CD_) reveals similar mosaic patterns in both cases, which comprise ordered and disordered bilayer regions resulting from the presence of the model TMD spanning the membrane (Figure S7). Particularly, palmitoyl modifications do not promote any specific ordering of POPC lipids in the vicinity of S-TMD with a small tendency towards a decrease in corresponding S_CD_ values. These observations are not at variance with data on quasi-harmonic (QH) entropy (Figure S4G) evaluated for each lipid molecule in the system. Like in the case of a pure POPC bilayer used as a reference, all systems with either unmodified or modified S-TMD trimer yielded a near-normal distribution of lipid entropies. TΔS as compared to pure POPC averaged −0.32 ± 1.19 kcal/mol and −0.36 ± 1.30 kcal/mol per lipid for unpalmitoylated and palmitoylated trimers, respectively, indicating minor perturbation of the lipid bilayer similar for modified and unmodified S-TMD.

## 3. Discussion

Ways in which the machinery effectuating viral fusion operates are impossible to fathom without taking into account the role of the membranes, both the host cell membrane and the viral envelope. This is due to the fact that the membrane is a major factor predetermining the structural and dynamic aspects of the behavior of the key players involved in fusion, these being the TMD as well as, at later stages, amphiphilic domains like HR1 and HR2. The latter only become employed at a certain point, but their contribution is of great importance. Furthermore, it is imperative to bear in mind that the listed fragments of the spike protein impact each other, enabling the virus to fulfill its potential in fusing the membranes completely.

When evoking the importance of understanding the molecular aspects of TMD organization and functioning, one shall have to think beyond its obvious role as an anchor, which ensures the correct positioning of the spike protein in the viral envelope. No less crucial are the subtle features of its structure and dynamics, which presumably enable the spike to effectuate conformational rearrangements varying in scale from local to global and determining the transition from the pre-fusion to post-fusion state. For example, the SARS-CoV spike, highly homologous to that in SARS-CoV-2 in the S2 subunit, showed much lower fusion activity upon introduction of point mutations in the aromatic-rich cluster. It was shown that when two out of three tryptophan residues in this cluster (corresponding to W1212, W1214, and W1217 in the SARS-CoV-2 spike) were substituted with phenylalanines, this resulted in a lack of a viral entry for SARS pseudo-particles, while replacement of only one of these Trp residues resulted in a residual level of entry [21]. Furthermore, the substitution of polar residues Thr1238 and Ser1239 located C-terminally to Ala led to a significantly lower level of acylation of the spike protein [22]. Acylation itself or the absence thereof had been demonstrated to bear an effect on the infectivity of both SARS-CoV [7] and SARS-CoV-2 [6] following substitutions of Ala for Cys residues 1235 and 1236 in the first cysteine cluster downstream of TMD (SARS-CoV-2 numbering). More or less similar effects have been reported in the SARS-CoV spike for point mutations G1201K (G1219 in SARS-CoV-2 spike) and V1210K (V1228 in SARS-CoV-2 spike) [21]. Finally, it has been experimentally demonstrated [23] that the GxxxG motif in the SARS-CoV spike (corresponding to 1219-GFIAG-1223 in SARS-CoV-2 spike) TMD is crucial to its trimerization, as substituting the first one of these glycines or both of them with isoleucine resulted in a dramatic reduction of oligomerization. (It is of note that these glycine residues lie on the helix-helix interface in our model *S*_*OPT*). To a considerable extent, this effect was observed at the level of the entire spike protein, not just isolated TMD. Shortly afterward, another study was published [24] in which substitution of the same residues with leucine failed to confirm the importance of the GxxxG motif, but the “second” glycine, corresponding to G1223 in the SARS-CoV-2 spike, proved to be crucial to the spike’s functioning. Taken together, these results demonstrate that the functioning of such complex machinery as the entire virion is sensitive to rather subtle modifications in TMD, which would presumably not be the case if TMD only served as a simple membrane anchor. Besides, it would seem highly likely that SARS-CoV-2’s S-TMD is organized following the same principles as the one in SARS-CoV, insofar as their TMD sequences are almost identical.

To propose a reliable configuration of TMD trimer corroborated by existing experimental observations, a comprehensive modeling framework was employed here with careful consideration of TMD boundaries and helix-helix association interfaces. Particularly, for the first time, homology modeling was done by adopting a combined algorithm of template selection, which relied on the search of TM α-helical homotrimer structures followed by singling out ones wherein the physicochemical properties of individual monomers bore a maximum resemblance to those in S-TMD. Such resemblance was recognized as the similarity between patterns of residues in their sequences possessing comparable physical and chemical properties, as well as the similarity between the distributions of hydrophilic, hydrophobic, and variability-prone properties on helix surface, also known as “dynamic molecular portraits” (see Section 2.4). Furthermore, the initial template-based models were additionally adjusted, taking into account the results of PREDDIMER [25] prediction of helix-helix packing to align the target protein and the template optimally. This was done due to the lack of homology of their sequences. Importantly, microsecond-long MD simulations in a model POPC membrane helped to improve the stability of the model via its iterative fine-tuning. This step was necessary, seeing that direct mounting of the S-TMD sequence even onto the best-suited template led, as we observed, to unstable behavior of the trimer in a water-lipid environment. This was due to the presence of hollow spaces (FV) in the trimer lumen, translating as suboptimal α-helix packing. These imperfections were identified in the course of MD and rectified manually, taking into account PREDDIMER results. The proposed model herein referred to as *S*_*OPT* eventually demonstrated a very high level of stability in independent runs of microsecond-range MD simulations. Interestingly, in most cases, models based on structures observed experimentally in membrane-like environments (detergent micelles, bicelles, etc.) do not demonstrate such stability in a POPC bilayer.

As opposed to tools for computational prediction of transmembrane dimer structure [25,26] that have proven to be adequate as a result of comparison against existing experimental models (including ones obtained post factum), efficacious and generalized methods of transmembrane trimer modeling have not yet been developed. Targeted sporadic attempts at such modeling have been made using regular symmetrical search, simulation annealing, and energy minimization [23] for SARS-COV spike, using PREDDIMER for the prediction of association interfaces for individual helical pairs and their combination for influenza haemagglutinins [27], or via multiscale simulations of self-assembly for individual TMDs of T-cell receptor [28]. The present study, on the other hand, demonstrates a consistent framework combining reliable prediction tools and MD simulations, which enables one to model noncovalently associated trimers of TM helices following a systematised procedure.

The proposed model was also shown to be stable in microsecond-range MD simulations, when two cysteine residues located immediately downstream of the TMD are palmitoylated. The modifications do not display significant constraining or “freezing” of POPC lipids in the vicinity of the trimer, while the general membrane perturbation effect is similar to that of the non-palmitoylated trimer, as is evidenced by the analysis of lipid order parameters and their configurational entropies. However, due to limitations of the MD simulations performed such as the single-component POPC bilayer and elevated temperature, fine-tuning of the lipid environment due to palmitoylation as well as its functional interpretation is not fully accessible in our modeling framework and should be further investigated. Apparently, such effects can be observed in lipid bilayers more complex in composition than POPC; some recent experimental data demonstrate the importance of this issue [29].

Seeing that the proposed TMD model has been created *in silico* and does not directly rely on experimental structural data, questioning its accuracy and level of similarity to other models would not be unjustified. As far as accuracy is concerned, only one structure of the TMD has been reported, which was obtained by NMR spectroscopy in lipid-detergent bicelles [4]. Helix–helix interfaces in this structure differ from those proposed in the present work (see Section 2.6). However, the NMR model has a number of features prompting one to wonder whether it corresponds to the native state of the TMD. Most importantly, its amino acid sequence contains four substitutions and one deletion, which could dramatically impact helix packing. Furthermore, bicelles as a membrane-mimicking environment might significantly differ from a lipid bilayer, especially in the vicinity of the TMD boundaries. In the study in question, the N-terminal portion of the peptide under investigation (corresponding to the aromatic-rich cluster) was not part of the TMD, and its structure was not resolved, while the segment spanning the membrane was short, only containing 16 residues (from L1218 to L1234). The authors observed a disordered fragment inclusive of residues 1212 to 1217, whereas this region in the SARS-CoV spike had previously been shown to assume a conformation redolent of helical [5] and to have an affinity for water/lipid interfaces. The omission of a cluster of aromatic residues capable of strongly interacting with each other and with the membrane environment could seriously affect the geometry of the entire TMD model. Our calculations indicate that the NMR model of the TMD does not remain compact in a POPC bilayer, to the likes of which it is probably poorly adapted. However, this circumstance cannot be regarded as proof of the conclusion drawn above. In our model, this fragment is close to the water/lipid interface, in agreement with experimental data indicating that this is where it tends to localize. Its propensity for existing in the helical conformation (in which it is rendered in our model) means that it could form one entity with residues located downstream, also in the helical conformation. That several free energy minima exist for bundles of α-helices has been previously shown for TM dimers (e.g., [30]) however, the key question in such cases is which one of these minima manifests itself in reality, i.e., for S-TMD, in the viral envelope similar in lipid composition to ERGIC.

The question of the structural/dynamic organization of the aromatic-rich region downstream of HR2, sometimes referred to as the pre-transmembrane domain [31], is very important because it serves as a kind of “hinge” that provides large-scale conformational transformations accompanying the transition from the pre- to the post-fusion state [32,33].Given the trimeric nature of this fragment and its richness in aromatic and positively charged residues, which can strongly interact with each other in different combinations, this domain most likely has high conformational plasticity. It is this quality that would allow it to perform the crucial mechanistic role in the process of merging the membranes of the virus and the target cell. Hence attributing a single precise structure to such domains would not seem to make a lot of sense. One could draw an analogy with known membrane proteins like receptor tyrosine kinases (RTK); such regions on the membrane-water interface can adopt various conformations, depending on the external forces coming into play at different moments during the effectuation of their function, as well as on their exposure to varying environmental conditions [34]. A key role in such events can be played by proline residues, whereof the peptide bond can adopt both cis- and trans-configurations, regulating the inclination of the N-terminal portion of the TM helix and the adjacent extracellular domain with respect to the membrane [35]. Note that in spike proteins, the membrane-proximal proline residue (Pro1213 in the spike of SARS-CoV-2) is highly conservative. There is a distinct sublineage of viruses [36], whereof spike proteins sport a lower than 28% identity to other coronaviral spikes, in which this position is occupied by alanine. However, as far as the apparent importance of proline in this position in other lineages goes, further experimental studies, in particular, using NMR spectroscopy, could shed light on its contribution to the fusion process.

Our model of S-TMD differs from other models thereof created as part of large-scale projects on full-length spike modeling, such as one based on a water-soluble coiled-coil template [8]. Interestingly, though, a model of the TMD built using HIV’s gp41 protein [11] is strikingly similar to ours. The authors proposed two versions of the TMD to use in a range of their full-length spike models, and both of them turned out to possess helix-helix interfaces very similar to those in *S*_*OPT*, although the gp41-based model is still less stable in a POPC membrane than the one proposed in the present work. Furthermore, the authors only palmitoylated Cys1236, but not Cys1235, identifying it as solvent-exposed. However, it is known that replacement of both these cysteines with alanine leads to a dramatic drop in the level of acylation, 80% for the spike protein of SARS-CoV-2 [6] and 56% for the spike of SARS-CoV [7]. While the input of palmitoylation of these two cysteines separately has not been verified, one could speculate that both of them might be solvent-exposed, as one-tenth of all cysteine residues in all palmitoylation clusters within the endodomain is less likely to account for 80% of palmitoylation compared to two-tenths thereof. Our model offers both Cys1235 and Cys1236 available for palmitoylation and positioned in such a way that, if palmitoylated, this does not affect the stability of the TMD trimer. Nevertheless, it is interesting that, as far as helix–helix interfaces within the TMD are concerned, our model and the one based on gp41 demonstrate a great level of convergence, having been obtained using completely different approaches. Like spike, gp41 is a class I fusion protein, and one cannot help wondering whether the convergence of these two models is due to features peculiar to viral fusion protein TMDs in addition to general principles of transmembrane α-helix packing.

Equally of note is the fact that the proposed trimer model can provide a direct structural explanation of the effect of some of the TM mutations mentioned above, as well as the role of the GxxxG motif (or just G1223), since, in our model, the residues in question belong to the interfaces of helix association (Figure S4F) and their substitution may modulate stability and configuration of the trimer.

Importantly, the *S*_*OPT* model has already been put to practical use to verify the MHP compatibility between the spike protein and hDHHC20, the enzyme conducting palmitoylation. In our model, the hydrophilic patch, formed by T1231, S1239, and the highly conserved throughout all geni of coronaviruses T1238, is solvent-exposed. T1238 and S1239 were shown to be crucial to acylation via mutagenesis, and it is thus hypothesized that the hydrophilic patch is part of the docking site for hDHHC20, which has a hydrophilic patch of its own next to where the fatty acid chain is harbored [22]. Our model, therefore, does not contradict experimental data on the accessibility of this potential docking site to the enzyme effectuating palmitoylation. It might, however, need to be further tested once extended to include the ectodomain or portions thereof, as their impact on the TMD in terms of mechanical tension might affect our model’s stability.

The purpose of the present study was not to model the way TMD operates, for instance, during the transition from the pre-fusion to the post-fusion state. This is the subject of further exploration, which will require gradual complication of the system, i.e., the addition of regions adjacent to the TMD. Most notably, one will have to consider domains HR1 and HR2 consisting of amphiphilic α-helices, major factors in spike refolding during fusion that eventually form the so-called “trimer of hairpins” made up of 6 helices and stabilizing the post-fusion state. It has already been demonstrated that HR regions possess an affinity for the membrane apart from an affinity for each other, HR1 much more so than HR2 [37]. It is thus possible and has indeed been speculated that such interactions facilitate viral fusion. Our model could be useful in the exploration of the behavior of these membrane-proximal fragments of the spike protein involved in fusion, most notably HR2, with recourse to computational tools similar to those employed in the present study. Such computational tools and our original modeling framework could potentially complement structure-based methods such as single-molecule force spectroscopy [38] (which has recently evolved to yield data in the microsecond range [39]), should they be used to study the packing mechanisms, conformational rearrangements and the subtleties of the energy landscape of TMDs.

## 4. Materials and Methods

### 4.1. Assessment of TMD Boundaries via Sequence Analysis and Monte Carlo Simulations with an Implicit Membrane

Initial guesses regarding the boundaries of the TMD were made using a set of bioinformatics tools applied to the spike sequence. Based on the predictions made using TMSEG [40] and TMPRED [41], as well as taking into account data on proposed TMD boundaries from UniProt entry P0DTC2 [42] and related publications, the peptide selected for a more detailed assessment of the structure and membrane localization of the S-TMD corresponded to residues 1208 to 1239 (QYIKWPWYIWLGFIAGLIAIVMVTIMLCCMTS). It was then examined via MC simulations with an implicit membrane model [43]. The starting structure of the TMD was constructed in the α-helical conformation (underlined in the aforementioned sequence). Peptide charges were assigned as if it remained at pH 7. At the beginning of MC calculations with an implicit membrane model, the peptide was arbitrarily placed outside the hydrophobic layer, or the so-called “hydrophobic slab.” To change the orientation of the peptide with respect to the slab during an MC simulation, a fragment of 21 dummy residues was attached to its N-terminus. These “virtual” residues did not contribute to the energy of the system. The first atom of the N-terminal dummy residue was always placed at the center of the slab and assigned coordinates (0,0,0). The conformational space of the peptide was explored via unrestrained MC search in a dihedral angles space using the modified FANTOM program [44] and our implicit solvation model for the membrane–mimicking heterogeneous water/cyclohexane environment [43]. The all-atom potential energy function was expressed as *E_total_* = *E_ECEPP/2_* + *E_solv._*, where the term *E_ECEPP/2_* includes van der Waals, torsion, electrostatic, and H-bonding contributions [45]. *E_solv._* is the solvation energy calculated as follows:

(1) $$\Delta E_{solv.}=\sum_{i=1}^{n}\Delta \sigma_{i}ASA_{i},$$

where Δσ_i_ and ASA are the atomic solvation parameter (ASP) and the solvent-accessible surface area (ASA) for atom i, respectively. ASP values for gas/water and gas/cyclohexane transfer were used as described elsewhere [46]. To cross the energy barriers between local minima, the adaptive-temperature schedule protocol [44] was employed. At each MC-step, the structures were subjected to 50 to 100 steps of conjugate gradient minimization. The ω dihedral angles were fixed (except those in dummy residues), and a spherical cutoff of 20 Å for nonbonded interactions was employed. Long-range electrostatic interactions were treated with distance-dependent dielectric permeability calculated as ϵ = 4 × r. The half-width of the membrane interface region and the slab thickness were 1.5 and 30 Å, respectively. Before the simulations, starting structures were subjected to 100–200 steps of unconstrained conjugate gradients minimization. For each peptide, several consecutive MC runs were carried out. At the initial stages of the MC-protocol, large structural changes were allowed via the sampling of several dihedrals, after which a more detailed conformational search with only a few varied dihedrals was performed. The starting structure for each MC run was the lowest-energy one obtained during the previous run. The set of resulting structures was analyzed using the following parameters: total energy, Z-coordinate of the center of mass of the TMD, secondary structure, depth of immersion of residues in the membrane, tilt angles of the helical segment with respect to the normal to the membrane plane (axis Z), hydrogen (H) bonding. Analysis of MC data was done using auxiliary programs specially written for this purpose. Along with other details of the computational protocol, they are described elsewhere [43,46].

### 4.2. Search for the Template for Homology Modelling of S-TMD

#### 4.2.1. Analysis of TM Sequences in Homotrimers of TMDs with Known Spatial Structure

Information on such homotrimers—candidate templates for S-TMD modeling—was gleaned from the OPM database [47]. Sequences of these TM fragments were then compared against that of S-TMD to identify patterns of residues with physicochemical properties in the TM sequence sufficiently similar to those in the fragment of interest in the spike. Positions of the following types of residues were taken into account: charged, polar, hydrophobic, small, and prolines (see Table S1).

#### 4.2.2. MHP Mapping and Helix–Helix Interface Analysis

Molecular hydrophobicity potential (MHP) maps were calculated as described elsewhere [48,49]. This was done for candidate templates and spike TM segment 1212 to 1234 built-in an ideal α-helical conformation. Helix-helix dimerization interfaces were predicted using the PREDDIMER web server (https://preddimer.nmr.ru) [25]. ASA values were calculated using DSSP [50]. A residue was considered to be located on the interface if the difference between its ASA values in a single helix and a helical oligomer was ≥25 Å^2^ (≥10 Å^2^ for glycine).

### 4.3. Modeling of S-TMD

Template-based modeling of the S-TMD was carried out in MODELLER9.19 [51]. The initial model was built based on the NMR structure of the TMD of TNFR-1 (PDB ID 7K7A) [18]. Small unordered sequences (Table 1) were added upstream and downstream of the TMD also using Modeller 9.19; the final model eventually included residues 1208–1238. *S_OPT*, the refined and better-packed model trimer, was built in Pymol 2.4.0 [52] based on MD-derived dimerization interfaces of tightly packed helices in the TNFR-1-based trimer (see Section 2.4). Tight clashes between residues were eliminated in UCSF Chimera 1.14 [53]. In all cases, point mutations were introduced in MODELLER9.19. Introducing palmitoyl modifications at C1235 and C1236, two different initial orientations of the short unordered peptide fragments downstream of L1234 were tested: (1) along the TMD axis and (2) parallel to the bilayer plane. In both cases, all six palmitoyl chains were oriented along the normal to the bilayer plane. Topology files for palmitoylated TMD monomers were generated using in-house software.

### 4.4. MD Simulations

On all occasions, the TMD fragment was aligned with the normal to the bilayer plane and was inserted into the system in such a manner as to span the membrane. MD simulations were performed using the GROMACS 2020.4 package [54] and the CHARMM36 force field [55,56,57,58,59]. An integration time step of 2 fs was used and 3D periodic boundary conditions were imposed. The spherical cut-off function (12 Å) was used to truncate van der Waals interactions. Electrostatic interactions were treated using the particle mesh Ewald (PME) method [60] (real space cutoff 12 and 1.2 Å grid with fourth-order spline interpolation). The TIP3P water model was used [61], and Na^+^ and Cl^−^ ion parameters for counter ions were implemented. Simulations were performed at 325 K temperature and 1 bar pressure maintained using the V-rescale [62] and the Parrinello–Rahman [63] algorithms with 0.5 and 5.0 ps relaxation parameters, respectively, and a compressibility of 4.5 × 10^−5^ bar^−1^ for the barostat. The protein, along with membrane lipids and solvent molecules, were coupled separately. Semi-isotropic pressure coupling in the bilayer plane and along the membrane normal was used in the simulations. Before the production runs, all systems were minimized over 2000 steps using a conjugate gradients algorithm, followed by heating from 5 K to 325 K over 50,000 steps, during which internal coordinates of the protein and ligand heavy atoms were restrained. Production runs were simulated for 0.5 to 1.0 μs depending on the system (see Table 1 and Table 2 for details). Bonds with an H atom were constrained via implementing LINCS [64].

### 4.5. Data Analysis

#### 4.5.1. MD Trajectory Analysis

All MD trajectories were processed using the *trjconv* utility from the GROMACS 2020.4 package to get the protein centered in the box, 3D periodic boundary conditions removed, and obtain an output frequency of 100 ps per frame. Coordinates were extracted using the *traj* utility, while RMSD and RMSF were calculated using the *rms* and *rmsf* utilities, respectively, all from the GROMACS package. Intermolecular contacts were calculated using PLATINUM [49] and other in-house software (e.g., [65]). Molecular editing and graphics rendering were performed using PyMOL v. 2.4.0 [52] and UCSF Chimera package v. 1.14 [53].

#### 4.5.2. Free Volume Calculation

For each trajectory frame, the trimer and surrounding molecules were oriented along the axes of a local coordinate system to calculate the 3D distribution of free volume (FV) available to probe spheres with a radius of 1.4 Å (the size of a water molecule or a CH_3_/CH_2_ group) within a regularly spaced rectangular mesh inside the trimer. Nodes of the mesh were considered free if their average occupancies over the analyzed frame sequence were lower than 0.5.

A regularly spaced rectangular mesh was placed so that the *X* axis of the local coordinate system (CS) would pass through the centers of mesh faces parallel to the YOZ plane, while the coordinate system origin would be on the lower YOZ face center. Local CS origin coincided with the center of mass (CoM base) of CA atoms in S-TMD residues 1234 and 1235 (residues 233 and 234 for TNFR-1). Its *X*-axis passed through the CoM of CA atoms in S-TMD residues 1211/1217 and 1212/1218 (depending on the model) or residues 211 and 212 in TNFR-1 (CoM top). The CA atom of residue 1234/233 in S-TMD/TNFR-1 chain A lay in the YOZ plane. The dimensions of the mesh were chosen empirically for the mesh to cover the TMD trimer lumen area (Figure S8). Nodes of the mesh were positioned with an increment of 0.5 Å along all axes. For each trajectory frame, the protein and surrounding molecules were oriented as described above, and free volume (FV) values were calculated.

Initially, all mesh vertices had a weight of 0. During FV estimation, each van-der-Waals (vdW) sphere associated with an atom was checked against nearby mesh vertices. All vertices lying inside a given vdW sphere were assigned weights equal to 1.

The data collected were used for the evaluation of the FV in the TMD trimer lumen, which was conducted as follows: all nodes where occupancy time was less than 50% were singled out, connected components of FV mesh nodes in 3D space were identified, and the largest component was selected. The node count of the largest component was then multiplied by the cell volume, and the size of its bounding box along the direction of the *X* axis was derived therefrom, providing estimated FV in the trimer lumen. FV fluctuations throughout MD trajectories were calculated in this manner by applying a moving average of over 200 frames.

#### 4.5.3. Membrane Response Evaluation

Conformational entropy for lipids was evaluated using the quasi-harmonic (QH) approach employing mass-weighted covariance matrices in Cartesian coordinates derived via the covar utility from the GROMACS package. The resulting eigenvalues were processed to calculate QH entropies as described previously [66]. For each system that underwent such analysis, QH entropies were calculated per lipid molecule. Conformational entropy values were evaluated relative to those in the “pure” POPC bilayer, which was simulated for 1 μs using the same MD protocol as for other systems (see Section 4.4). For entropy calculation, the first 0.25 μs of each trajectory were omitted to avoid artifacts related to system equilibration.

Carbon-deuterium order parameters (S_CD_) for the acyl chains in POPC molecules were calculated as described elsewhere [67], while S_CD_ maps were created using in-house software. To this end, S_CD_ parameters for atoms in lipid acyl chains were calculated for all frames in the MD trajectory prepared with recourse to the *−fit rotxy + transxy* setting in the trjconv utility from the GROMACS 2020.4 package. Coordinates of the atoms were used to distribute S_CD_ values over a two-dimensional 50 × 50 units grid on the membrane plane equalling in size to the simulation cell dimensions. The data were averaged over the trajectory and used to plot the S_CD_ maps for each bilayer leaflet.

## Figures and Tables

**Table 1:** Model trimers tested in the present study.

Name	Sequence
Created via template-based modelling	
*S*_*TNFR1*	1208-QYIK**WPWYIWLGFIAGLIAIVMVTIML**CCMT-1238
The template used to build the TMD model (TNFR-1)	
*TNFR1*_*TMD*	209-GTT**VLLPLVIFFGLALLSLLFIGLA**YRYQR-238
Built iteratively via the adjustment of the MHP portraits in *S*_*TNFR1*	
*S*_*OPT*	1208-QYIK**WPWYIWLGFIAGLIAIVMVTIML**CCMT-1238
Based on the recently published NMR structure (PDB ID 7LC8)	
*S*_*NMR*	1217-W**LGFIAGLIAIVLVTILL**SST-1237
*S*_*NMR+*	1214-WYIW**LGFIAGLIAIVLVTILL**SSTSCC-1240
*S*_*NMR-WT*	1217-W**LGFIAGLIAIVMVTIML**CCM-1237
*S*_*OPT* with the mutations present in the recent NMR structure (PDB ID 7LC8)	
*S*_*OPT-LC*	1208-QYIK**WPWYIWLGFIAGLIAIVLVTILL**CCMT-1238
*S*_*OPT-LS*	1208-QYIK**WPWYIWLGFIAGLIAIVLVTILL**SSMT-1238
*S*_*OPT* with palmitoyl tails appended at C1235 and C1236	
*S*_*OPT-PLM*	1208-QYIK**WPWYIWLGFIAGLIAIVMVTIML**CCMT-1238

In all cases (except *S*_*NMR* and *S*_*NMR-WT*), small disordered fragments were added upstream and downstream of the TMD (TMD residues are bold-typed). Acylation sites in palmitoylated models are underlined. Substitutions in the experimental NMR trimer [4] compared to the wild-type spike protein are highlighted in yellow.

**Table 2:** MD trajectories calculated in the present study.

System Composition	MD Length (ns)	No. of Replicas
***A POPC bilayer***		
POPC_450_/Water_26276_	1000	1
***TNFR1_TMD in a lipid bilayer***		
Protein/POPC_475_/Water_42057_/Cl^−^_6_	500	1
***S_TNFR1 in a lipid bilayer***		
Protein/POPC_483_/Water_42060_/Cl^−^_3_	500	4
***S_OPT in a lipid bilayer***		
Protein/POPC_485_/Water_42098_/Cl^−^_3_	1000	2
***Structures based on the experimentally observed trimer (PDB ID 7LC8) in a lipid bilayer***		
*S_NMR*/POPC_488_/Water_42196_	500	1
*S_NMR+*/POPC_485_/Water_42148_	500	1
*S_NMR-WT*/POPC_486_/Water_42198_	500	1
***S_OPT with the mutations present in the experimentally observed trimer (PDB ID 7LC8) in a lipid bilayer***		
*S_OPT-LC*/POPC_483_/ Water_42092_/Cl^−^_3_	1000	1
*S_OPT-LS*/POPC_484_/Water_42117_/Cl^−^_3_	1000	1
***S_OPT palmitoylated at C1235 and C1236***		
Protein/POPC_480_/Water_42084_/Cl^−^_3_	1000	1
Protein/POPC_474_/Water_42120_/Cl^−^_3_	1000	1

The number of molecules/ions of each type in the system, if above 1, is presented in subscript. See Table 1 for more details on the peptides studied.

## Data Availability

The model of SARS-CoV-2 spike TMD proposed in the present study is available upon request in PDB.

## Supplementary Materials

The following supporting information can be downloaded at: https://www.mdpi.com/article/10.3390/ijms23169221/s1.

## Abbreviations

The following abbreviations are used in this manuscript:

TM	transmembrane
TMD	transmembrane domain
S-TMD	spike transmembrane domain
FV	free volume
MC	Monte Carlo
MHP	molecular hydrophobicity potential.
