# Supplementary material for: A Uniquely Stable Trimeric Model of SARS-CoV-2 Spike Transmembrane Domain
Source: Int J Mol Sci. 2022 Aug 17;23(16):9221. doi: 10.3390/ijms23169221 (PMC9409440; doi:10.3390/ijms23169221)
Supplement: Supplementary file 1 [file ijms-23-09221-s001.zip › ijms-1844503-supplementary.pdf]

A Uniquely Stable Trimeric Model of SARS-CoV-2 Spike Transmembrane Domain

SUPPLEMENTARY INFORMATION

Aliper E.T.[1], Krylov N.A.[1,2], Nolde D.E.[1,2], Polyansky A.A.[1,3], Efremov R.G.[1,2,4] *

[1] Shemyakin-Ovchinnikov Institute of Bioorganic Chemistry, Russian Academy of Sciences, 16/10 Miklukho-Maklaya St., Moscow 117997, Russia;
[2] National Research University Higher School of Economics, Moscow 101000, Russia
[3] Department of Structural and Computational Biology, Max Perutz Labs, University of Vienna, Campus Vienna BioCenter 5, A-1030 Vienna, Austria
[4] Moscow Institute of Physics and Technology (State University), Dolgoprudny, Moscow region, 141701, Russia
* Correspondence: efremov@nmr.ru

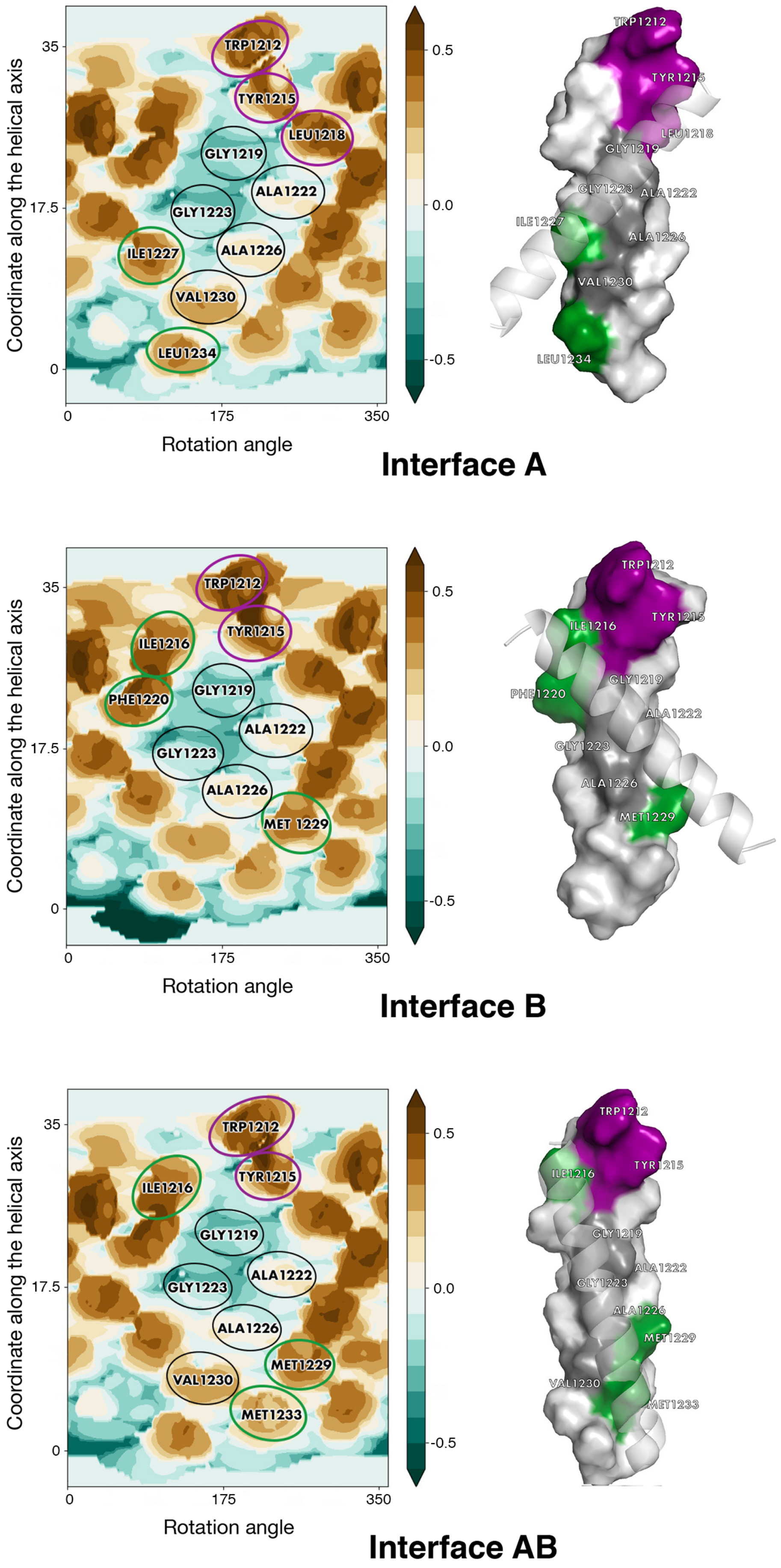

Figure S1. Dimerization interfaces predicted for S-TMD. Dimerization interfaces were predicted for an ideal helix with a sequence corresponding to S-TMD residues 1212-1234. In the MHP maps, identical positions are encircled in purple, conservative and semi-conservative residues are encircled in green, non-conservative residues present on the helix/helix interface are encircled in black. Cylindrical projection of the surface MHP distribution is used. Axis values correspond to the rotation angle around the helical axis and the distance along the latter, respectively. MHP scale (in logP octanol-1/water units) is presented on the right. The maps are coloured in accordance with the MHP values (Efremov et al., 1992), from teal (hydrophilic areas) to brown (hydrophobic ones). In the 3D models corresponding to each interface, identical and (semi-)conservative positions coloured in accordance with the same colour code as in the MHP maps, non-conservative residues located on the interface are coloured grey; surface representation of one of the helices is offered, while the second helix is shown in cartoon representation and is semi-transparent.

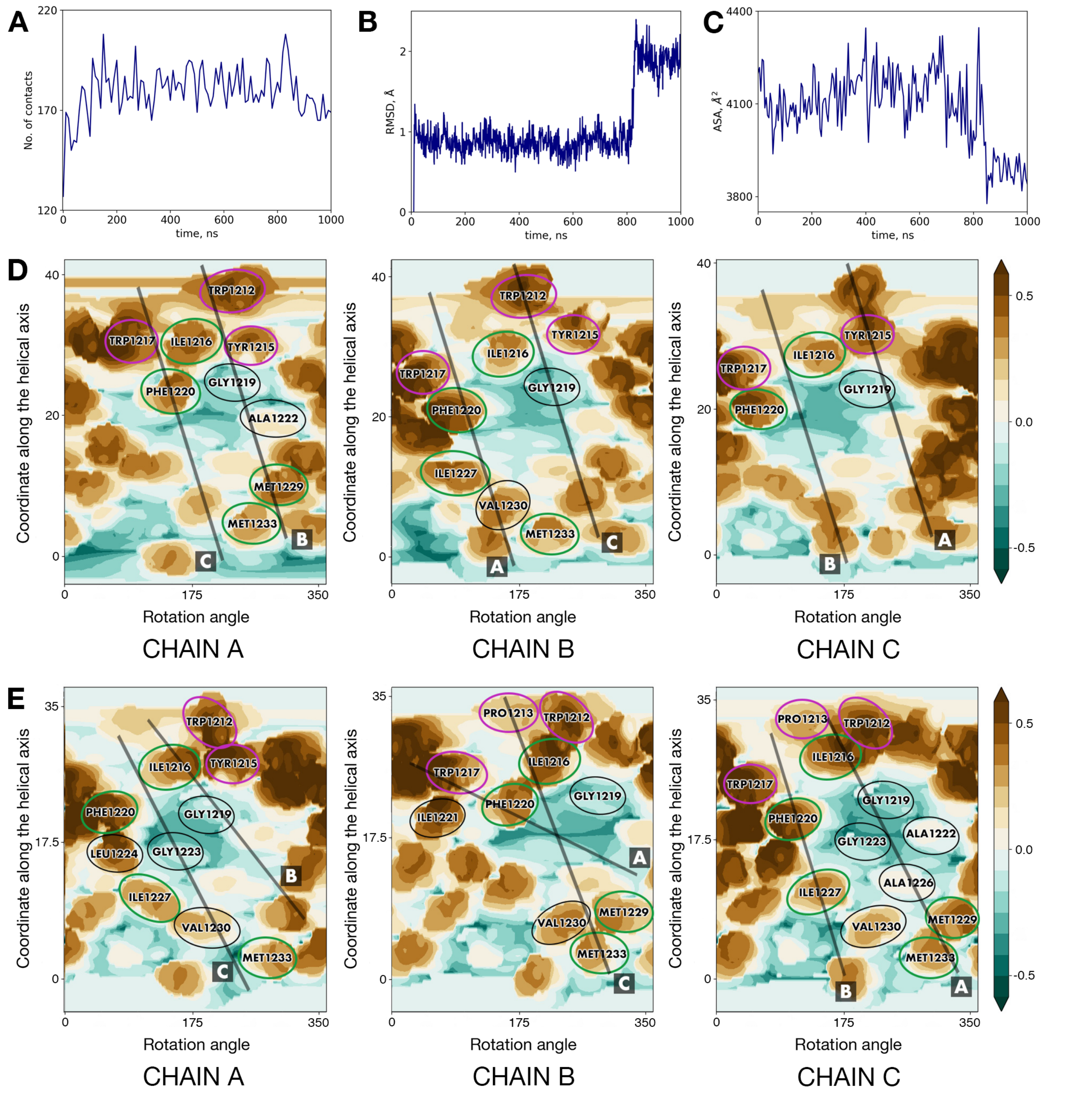

Figure S2. Performance of *S_TNFR1* in a POPC bilayer. (A) Protein/lipid contacts for the side chains in (originally) lumen-facing residues (1212, 1216, 1219, 1220, 1222, 1223, 1226, 1227, 1229, 1230, 1233) in *S_TNFR1*. **(B)** RMSD dynamics calculated for the backbone atoms of residues 1212-1234 in the two chains of *S_TNFR1* (A and C) that went on to form a highly stable dimer. The state registered at 9ns, the first frame when it was detected, was used as the reference frame for RMSD calculation. **(C)** Solvent-accessible surface area (ASA) for the same dimer made up by chains A and C. **(D)** Helix/helix interfaces in *S_TNFR1* at 0 ns. **(E)** Helix/helix interfaces in *S_TNFR1* at 200ns. Identical positions are encircled in purple, conservative and semi-conservative residues are encircled in green, non-conservative residues present on the helix/helix interface are encircled in black. In the MHP map for each chain, schematic projections of the other two chains are shown as thick black lines and are designated by letters A to C. For other details see legend to Fig. S1.

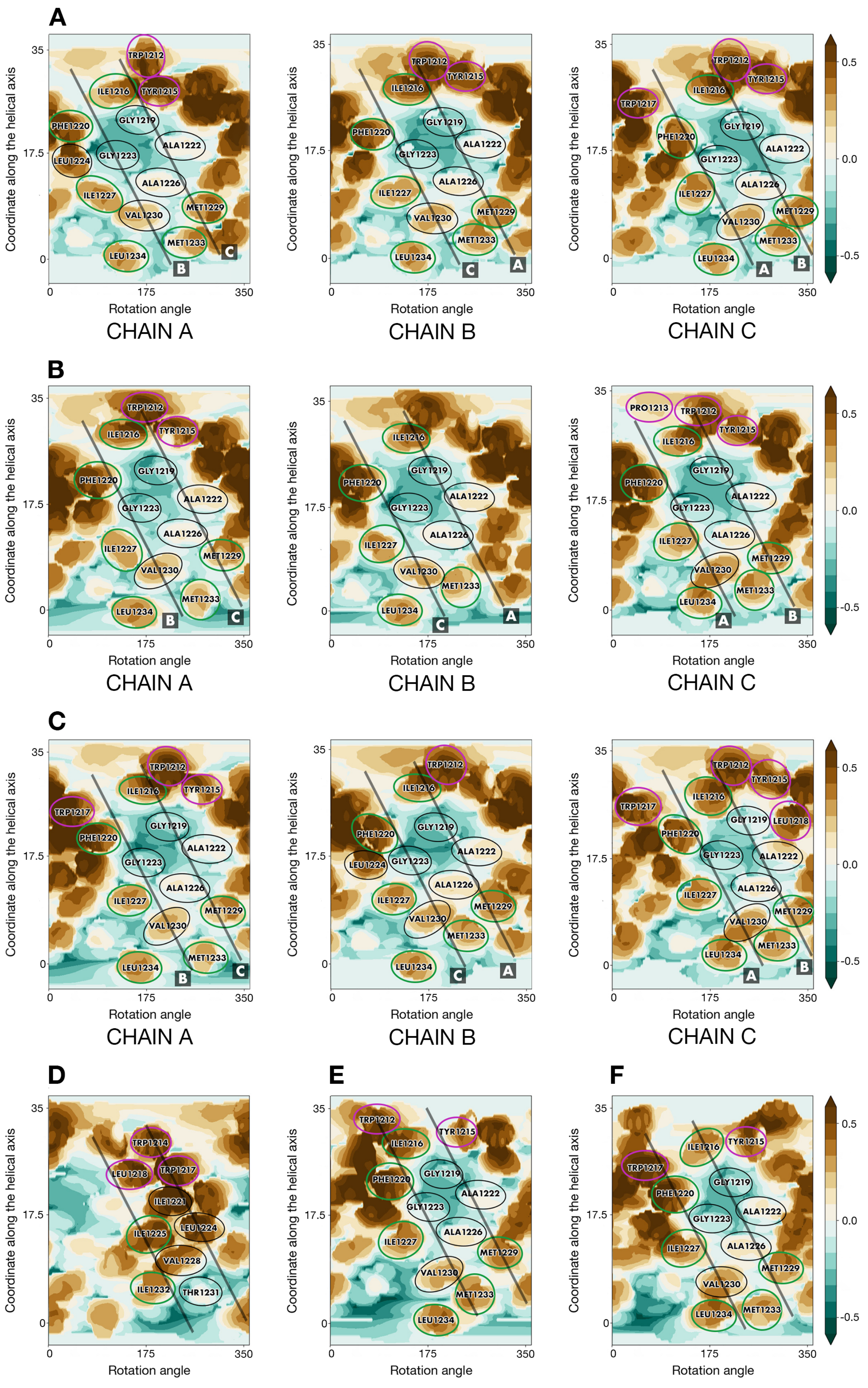

Figure S3

Figure S3. Helix/helix interfaces in *S_OPT* and certain alternative models on MHP maps. (A) *S_OPT* after energy minimisation. **(B)** *S_OPT* at 1μs in trajectory run1. **(C)** *S_OPT* at 1μs in trajectory run2. **(D-F)** Models created by Casalino et al. (2020) (D) and two versions (E and F) of the S-TMD by Woo et al. (2020) built using HIV's gp41 as template. Identical positions are encircled in purple, conservative and semi-conservative residues are encircled in green, nonconservative residues present on the helix/helix interface are encircled in black. In the MHP map for each chain, schematic projections of the other two chains are shown as thick semi-transparent black lines and, where appropriate, are designated by letters A to C. For other details see legend to Fig. S1.

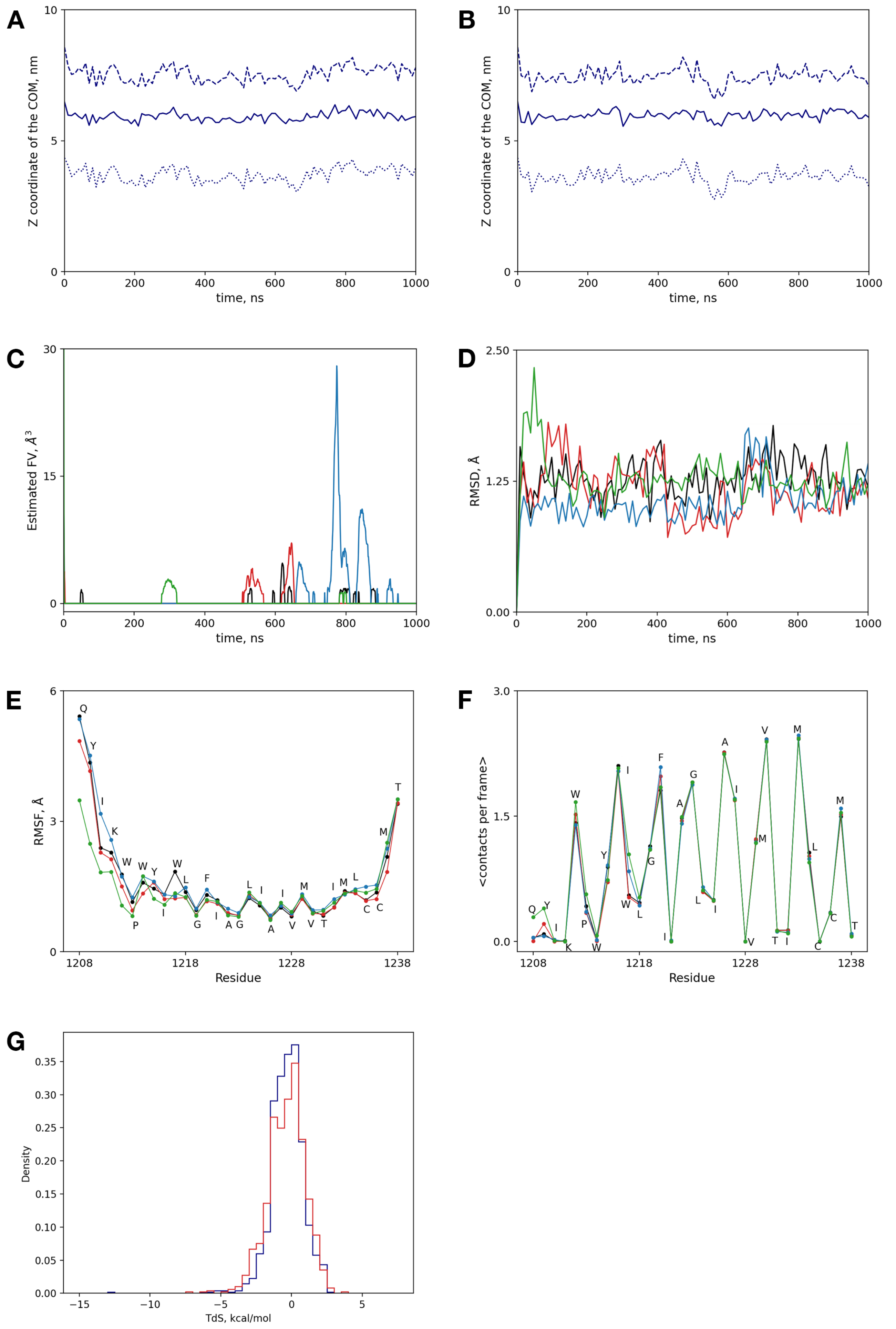

Figure S4

Figure S4. Performance of *S_OPT* and *S_OPT-PLM* in a POPC bilayer. (A-B) Z coordinate of the centre of mass (COM) of *S_OPT* residues 1212-1234 in run1 (A) and run2 (B), indicating trimer localization (solid lines) in the membrane with respect to the COM of the P atoms of the upper (dashed lines) and lower (dotted lines) leaflets of the POPC bilayer. **(C)** Estimated free volume (FV) in the lumen in *S_OPT* (black and red) and *S_OPT-PLM* (blue and green) trimers over the course of their respective trajectories. **(D)** RMSD calculated for the backbone atoms of residues 1212-1234 in *S_OPT* (black and red) and *S_OPT-PLM* (blue and green). Frame 0 was used as the reference structure in all cases. **(E)** RMSF values for individual residues in *S_OPT* (black and red) and *S_OPT-PLM* (blue and green). **(F)** Involvement of individual residues in helix/helix non-covalent interactions in palmitoylated (blue and green) and unpalmitoylated (black and red) variants of *S_OPT*. (G) Normalised histograms of TΔS distribution for POPC molecules in systems containing *S_OPT* (blue) and *S_OPT-PLM* (red) as compared to a "pure" POPC bilayer.

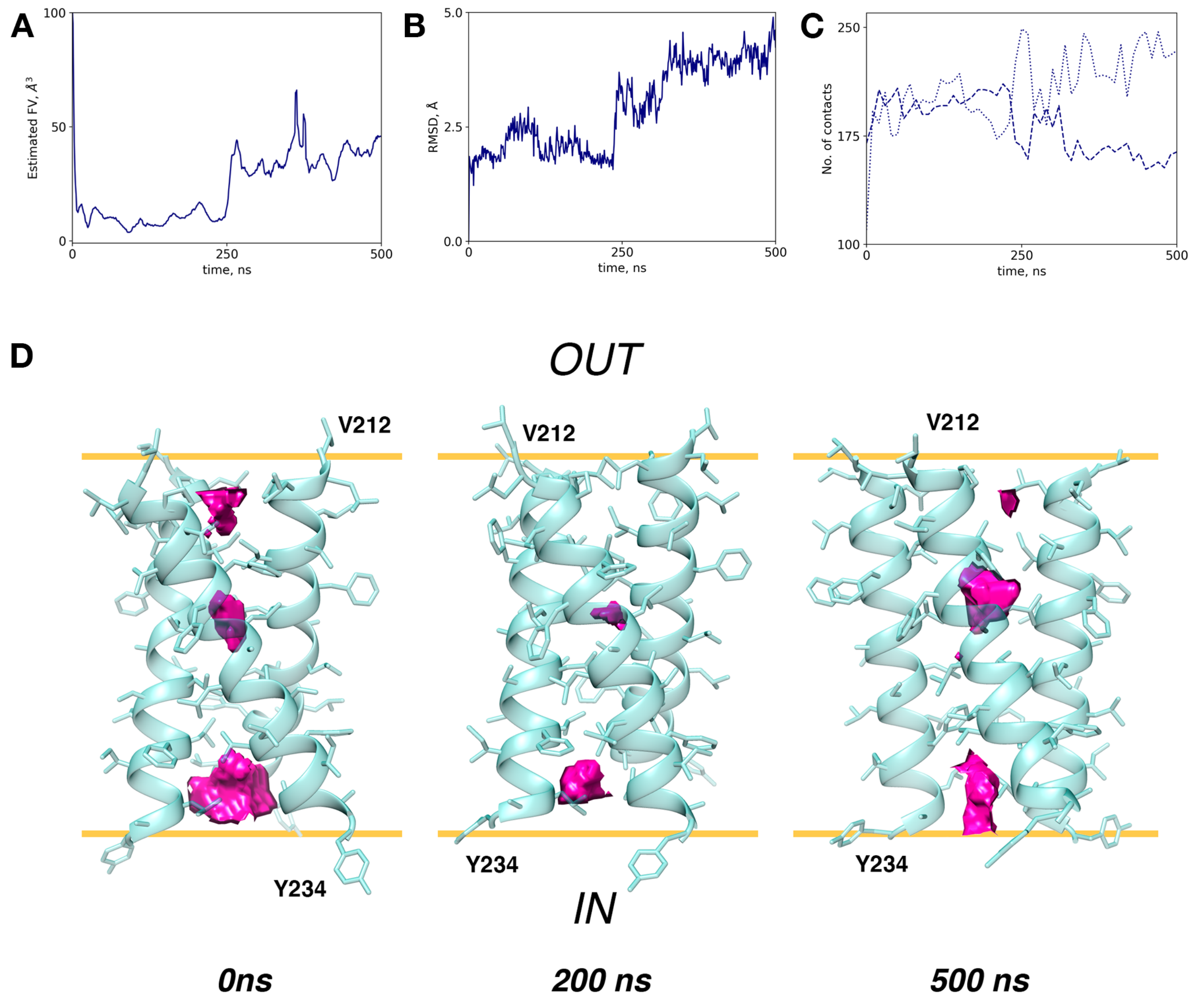

Figure S5 (above). Results of the MD simulation of TNFR-1 TMD in a POPC bilayer. (A) Estimated free volume inside TMD during an MD simulation in a POPC bilayer. **(B)** RMSD for the backbone atoms (residues 212 to 233). **(C)** Protein/protein (dashed line) and protein/lipid (dotted line) contacts for the side chains constituting the interface (214, 217, 218, 219, 221, 222, 224, 225, 228, 229, 232). **(D)** Visualisation of estimated free volume (pink) inside the TMD at 0 ns, 200 ns and 500 ns (averaging over 200 frames not applied). 3D models are shown in cartoon/stick representation. Approximations of carbonyl/ester oxygen planes are shown as yellow lines, while the inside of the cell and the extracellular space are labelled "IN" and "OUT", respectively.

Figure S6 (right). Performance of NMR-based models and mutant forms in a POPC bilayer. (A-B) Contacts made by the side chains of I1221, I1225, L/M1229 and L/M1233 with lipids (A) and with the same side chains in other helices of the homotrimer (B) calculated for trajectories *S_NMR* (black), *S_NMR+* (red) and *S_NMR-WT* (blue). **(C-D)** RMSD of the backbone atoms of the proposed TMD (residues 1218-1234) and estimated free volume inside the lumens model trimers *S_NMR* (black), *S_NMR+* (red), *S_NMR-WT* (blue) and *S_OPT* (green) over the course of MD simulations. **(E)** Visualisation of free volume in model trimers *S_NMR*, *S_NMR+* and *S_NMR-WT*. 3D models are shown in cartoon/stick representation, and free volume is rendered as pink slabs (averaging over 200 frames not applied). Approximations of carbonyl/ester oxygen planes are rendered as yellow lines, the inside of the virion and the external environment are labelled "IN" and "OUT", respectively. **(F)** Estimated free volume in the lumen of *S_OPT-LC* (dashed line) and *S_OPT-LS* (dotted line) over the course of their respective trajectories. **(G)** RMSD calculated for the backbone atoms of residues 1212-1234 in model trimers *S_OPT-LC* (dashed line) and *S_OPT-LS* (dotted line); frame 0 was used as the reference structure in both cases.

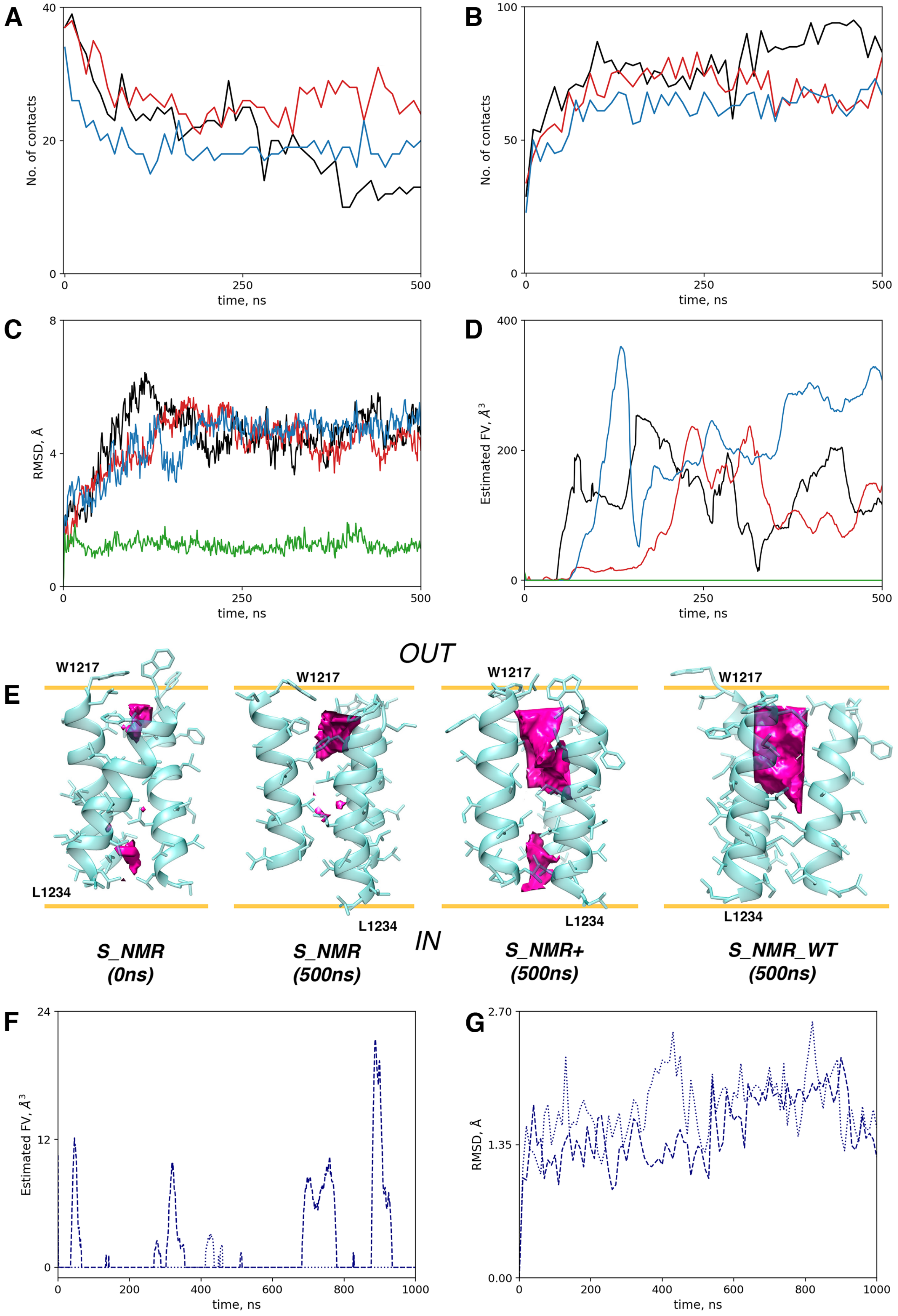

Figure S6

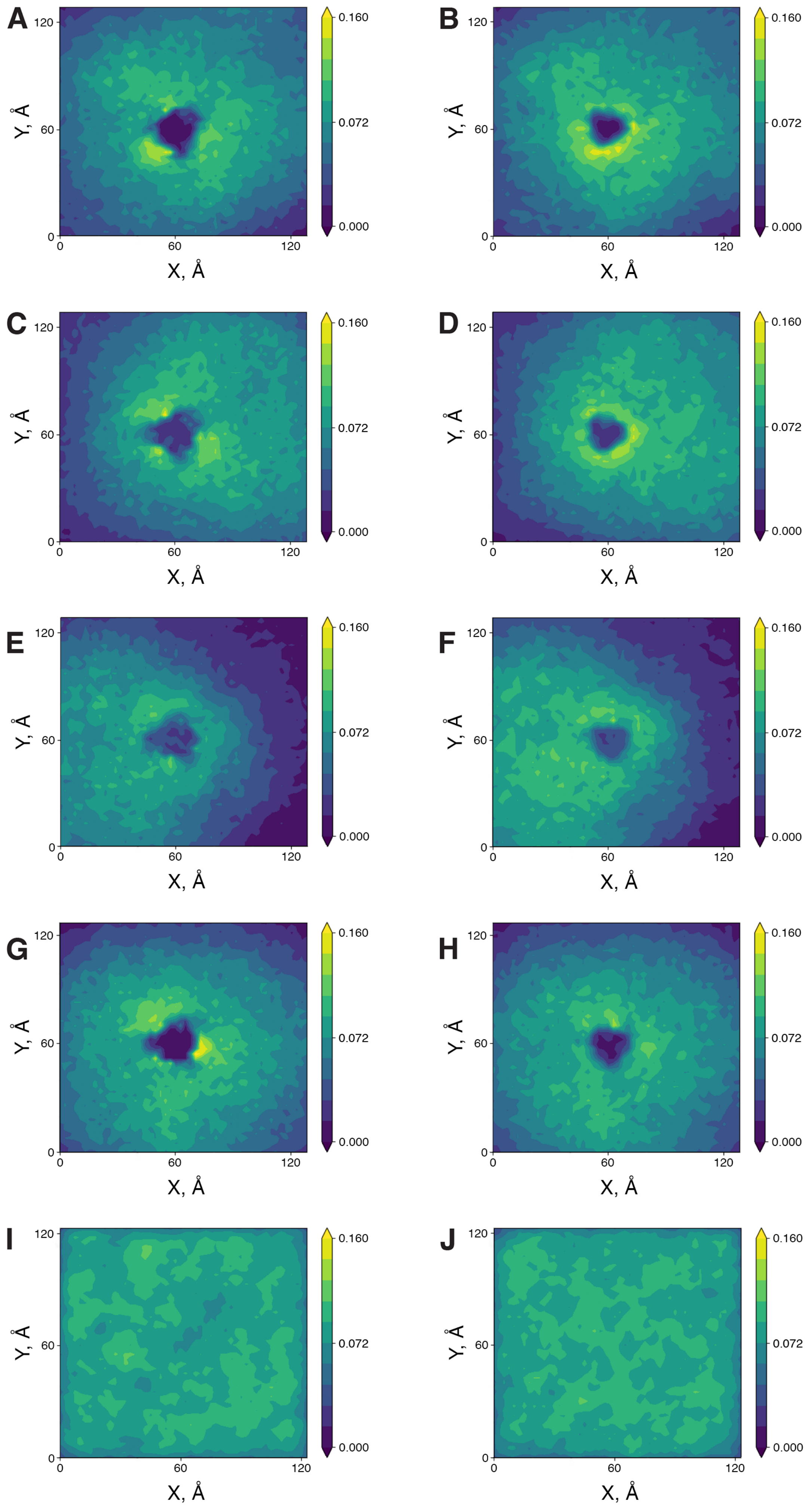

Figure S7

Figure S7. Order parametre maps plotted for model POPC bilayers. (A-B) *S_OPT* run 1 top leaflet (A) and bottom leaflet (B). (C-D) *S_OPT* run 2 top leaflet (C) and bottom leaflet (D). **(E-F)** *S_OPT-PLM* run 1 top leaflet (E) and bottom leaflet (F). **(G-H)** *S_OPT-PLM* run 2 top leaflet (G) and bottom leaflet (H). **(I-J)** A "pure" POPC bilayer top leaflet (I) and bottom leaflet (J). Maps were plotted over a period from 50ns to 1000ns for each trajectory. All bilayers are oriented in such a way that the normal to the bilayer plane is parallel to the Z axis, and the axes in maps, labelled X and Y, are oriented along eponymous axes in each system. An S_{CD} order parameter colour scale is presented on the right of each map.

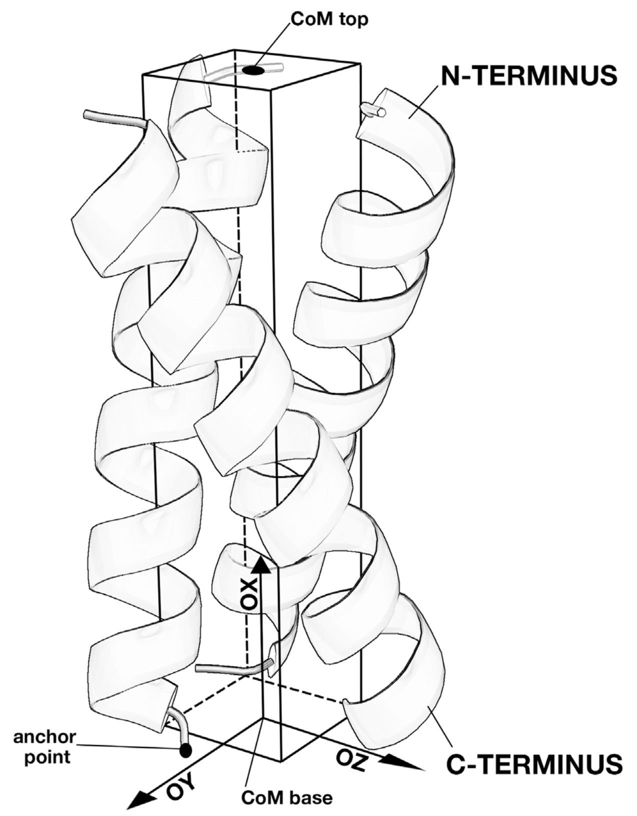

Figure S8

Figure S8. Mesh applied to calculate free volume (FV) inside a helical trimer. The helices are shown in ribbon representation. The confines of the mesh are shown as black lines. The CoM top and anchor point are shown as black dots. Axes of the local coordinate system originating at CoM base are shown conventionally and labelled accordingly. See section 4.5.2 for details.

SUPPLEMENTARY TABLES

Table S1. Amino acid sequences of TMDs for which structural data are available.

Protein	PDB ID	Sequence
GP41 (HIV)	*5JYN*	WLWYIRIFIIIVGSLIGLRIVFAVLSLV
LAMP-2	*2MOM*	FLVPIAVGAALAGVLILVLLAYFIGL
Human TNFR-1	*7K7A*	VLLPLVIFFGLALLSLLFIGLAY
TYRO protein tyrosine kinase-binding protein	*4WOL*	VSPGVLAGIVVGDLVLTVLIALAVYFL
Toll-like receptor 3	*2MKA*	LFFMINTSILLIFIFIVLLIHF
Murine TNF receptor 6	*2NA6*	LWLLTILVLLIPLVFIY
Vascular endothelial growth factor receptor 2	*2MET*	LEIIILEGTAVIAMFFWLLLVIIL

Hydrophobic residues are unhighlighted, while proline, small, charged and polar non-charged residues are highlighted in coral, blue, turquoise and yellow, respectively.

Table S2. Intermolecular protein-protein (P/P) and protein-lipid (P/L) contacts[a] in *S_OPT* and its derivatives over the course of MD simulations.

Trimer	P/P	P/L[b]
S_OPT run1	199±5	126±9
S_OPT run2	199±6	124±10
S_OPT-LC	191±6	130±9
S_OPT-LS	197±6	127±9
S_OPT-PLM run1	197±7	113±10
S_OPT-PLM run2	199±6	114±10

[a]: The number of pairs of atoms engaged in intermolecular contacts formed by the side chains of S_TMD's lumen-facing residues (1212, 1216, 1219, 1220, 1222, 1223, 1226, 1227, 1229, 1230, 1233, 1234) was calculated.
[b]: including protein-bound palmitoyl chains, when present.
